# Identification and expression profile of the *SMAX/SMXL* family genes in chickpea and lentil provide important players of biotechnological interest involved in plant branching

**DOI:** 10.1007/s00425-023-04277-y

**Published:** 2023-11-15

**Authors:** Marcos Fernando Basso, Felice Contaldi, Fabrizio Lo Celso, César Milton Baratto, Maria Fatima Grossi-de-Sa, Giampaolo Barone, Antonio Ferrante, Federico Martinelli

**Affiliations:** 1 Department of Biology, University of Florence, 50019 Sesto Fiorentino, Italy; 2 Department of Physics and Chemical, University of Palermo, Viale Delle Scienze, Edificio 17, 90128 Palermo, Italy; 3 University of Western Santa Catarina, Biotechnological Center, UNOESC, Videira, SC 89566-252 Brazil; 4 Embrapa Genetic Resources and Biotechnology, Brasília, DF 70297-400 Brazil; 5 Department of Biological, Chemical and Pharmaceutical Sciences and Technologies, University of Palermo, Viale Delle Scienze, Edificio 17, 90128 Palermo, Italy; 6 Department of Agricultural and Environmental Sciences, University of Milan, Via Festa del Perdono, 20122 Milan, Italy

**Keywords:** Abiotic stress, Biotechnological assets, Branching, BRC1, Karrikins, Legumes, Plant architecture, Strigolactones, Transcription factor

## Abstract

**Main conclusion:**

*SMAX/SMXL* family genes were successfully identified and characterized in the chickpea and lentil and gene expression data revealed several genes associated with the modulation of plant branching and powerful targets for use in transgenesis and genome editing.

**Abstract:**

Strigolactones (SL) play essential roles in plant growth, rooting, development, and branching, and are associated with plant resilience to abiotic and biotic stress conditions. Likewise, karrikins (KAR) are “plant smoke-derived molecules” that act in a hormonal signaling pathway similar to SL playing an important role in seed germination and hairy root elongation. The *SMAX/SMXL* family genes are part of these two signaling pathways, in addition to some of these members acting in a still little known SL- and KAR-independent signaling pathway. To date, the identification and functional characterization of the *SMAX/SMXL* family genes has not been performed in the chickpea and lentil. In this study, nine *SMAX/SMXL* genes were systematically identified and characterized in the chickpea and lentil, and their expression profiles were explored under different unstressless or different stress conditions. After a comprehensive in silico characterization of the genes, promoters, proteins, and protein-protein interaction network, the expression profile for each gene was determined using a meta-analysis from the RNAseq datasets and complemented with real-time PCR analysis. The expression profiles of the *SMAX/SMXL* family genes were very dynamic in different chickpea and lentil organs, with some genes assuming a tissue-specific expression pattern. In addition, these genes were significantly modulated by different stress conditions, indicating that *SMAX/SMXL* genes, although working in three distinct signaling pathways, can act to modulate plant resilience. Most *CaSMAX/SMXL* and partner genes such as *CaTiE1* and *CaLAP1*, have a positive correlation with the plant branching level, while most *LcSMAX/SMXL* genes were less correlated with the plant branching level. The *SMXL6*, *SMXL7*, *SMXL8*, *TiE1*, *LAP1*, *BES1*, and *BRC1* genes were highlighted as powerful targets for use in transgenesis and genome editing aiming to develop chickpea and lentil cultivars with improved architecture. Therefore, this study presented a detailed characterization of the *SMAX/SMXL* genes in the chickpea and lentil, and provided new insights for further studies focused on each *SMAX/SMXL* gene.

**Supplementary Information:**

The online version contains supplementary material available at 10.1007/s00425-023-04277-y.

## Introduction

Strigolactones (SL) are phytohormones that play essential roles in plant growth, rooting, development, and branching, and act to improve plant resilience (Yang et al. 2020; Li et al. 2022; Zhang et al. 2022). For the SL biosynthesis, all-trans-β-carotene are metabolized by the DWARF27 (D27) enzyme to produce 9′-cis-β-carotenoid, and the next steps of this pathway have the involvement of carotenoid cleavage dioxygenase (CCD) enzymes for synthesis of 9′-cis-β-10′-carotenal, carlactone, and 5-deoxylstrigol (Lopez-Obando et al. 2015; Wang et al. 2015; Bennett et al. 2016; Wallner et al. 2017; Sun et al. 2022). In the SL-dependent signaling pathway, the DWARF14 (D14) protein acts as an SL receptor, which binds and changes its molecular structure (Yao et al. 2016). D14 and SL complexes bind to D53/Suppressor of MAX2 1-Like (SMXL; formally named in *Arabidopsis thaliana* as AtSMXL6, AtSMXL7, and AtSMXL8) proteins in the nucleus, then recruit SCF^MAX2^ (SCF MORE AXILLARY GROWTH 2) protein to form D14-SL-SCF^MAX2^-D53/SMXLs complex (Bennett et al. 2016). SL promotes D53/SMXL ubiquitination, while the 26S proteasome specifically recognizes D53/SMXL proteins and directs to degradation, unlocking SL-dependent signal transduction and releasing BRANCHED 1 (BRC1) transcription factor (Zhou et al. 2013; Wang et al. 2015; Bennett et al. 2016). In this way, since D53/SMXL proteins are not degraded, SL-dependent signal transduction is inhibited while branching and tillering are promoted (Zhou et al. 2013; Zhang et al. 2022). Thus, D53/SMXL proteins are the final target proteins of this signaling pathway. Therefore, SL and D53/SMXL play a critical role in BRC1-mediated regulation of shoot branching and plant elongation (Zhao et al. 2015).

Likewise, karrikins (KAR) are butenolide molecules derived from plant smoke that act in a signaling pathway similar to the SL pathway (Bennett et al. 2016; Yang et al. 2019). In the KAR-dependent signaling pathway, KARRIKIN-INSUSCEPTIBLE2 (KAI2) protein acts as a KAR receptor (Villaécija-Aguilar et al. 2019). Subsequently, the KAR-KAI2 complex interacts with SCF^MAX2^ and SUPPRESSOR OF MAX2 1 (AtSMAX1/SMXL1), thus triggering ubiquitylation and targeting the AtSMAX1/SMXL1 and AtSMXL2 proteins for degradation (Carbonnel et al. 2020; Wang et al. 2020). Consequently, KAR-responsive genes, such as *ACC synthase 7*, which catalyzes ethylene biosynthesis, are transcriptionally up-regulated (Carbonnel et al. 2020). So, the KAR-dependent signaling pathway regulates seed germination and hairy root elongation (Wallner et al. 2017; Villaécija-Aguilar et al. 2019; Carbonnel et al. 2020). In addition, AtSMXL2 protein is also supposed to act in both SL- and KAR-dependent signaling pathways (Wang et al. 2020). Meanwhile, AtSMXL3, AtSMXL4, and AtSMXL5 proteins act in a yet unknown SL- and KAR-independent signaling pathway regulating phloem formation (Wallner et al. 2017). Given these previous studies, the biological importance of the SL- and KAR-dependent or -independent signaling pathways for plant development and resilience was determined in model plants. However, to date, little information about *SMAX/SMXL* family genes and their expression profile was generated from chickpea and lentil.

Chickpea (*Cicer arietinum* L.) and lentil (*Lens culinaris* Medik) are crops of outstanding importance for human food worldwide (Landi et al. 2021). The chickpea is a self-pollinated diploid, dicotyledonous, with a 738 Mb genome size organized in eight chromosomes (2*n* = 16) and up to 28,200 annotated genes (Varshney et al. 2013). In contrast, lentil is a self-pollinated diploid, dicotyledonous, with a 3.69 Gb genome size organized in seven chromosomes (2*n* = 14) and 58,243 annotated genes (Ramsay et al. 2023). Several germplasm banks with a high number of accessions, lines, and cultivars with high phenotypic variability are available for these two legumes. In particular, several cultivars of chickpea and lentil have a high number of branches and the absence of a typical dominant stem (Singh et al. 2019b; Silva-Perez et al. 2022). These intrinsic features related to plant architecture make it difficult to manage the chickpea and lentil crops in the field. For example, making mechanized harvesting more difficult and increasing lodging and susceptibility to pathogens (Tripathi et al. 2022). So, understanding the molecular mechanisms that orchestrate plant branching is essential for the development of chickpea and lentil cultivars with an improved architecture (Koul et al. 2022).

In this study, were systematically identified and characterized nine *SMAX/SMXL* family genes in the chickpea and lentil. The orthologous genes in the chickpea and lentil were identified using as reference *AtSMAX/SMXL* genes from *A. thaliana*. Subsequently, evolutionary relationships, features of sequences, the basic structure of genes, chromosomal localization of genes, *cis*-regulatory elements in promoter sequences, conserved motifs and domains in protein sequences, protein–protein interaction network, and three-dimensional (3D) structures of the SMAX/SMXL proteins were successfully performed and their biological implications were discussed. Expression profiles of the *SMAX/SMXL* genes in different organs of chickpea and lentil unstressed plants and under abiotic and biotic stress conditions were performed using a meta-analysis approach from RNAseq datasets. Finally, expression profiles of all identified *SMAX/SMXL* genes in the chickpea and lentil contrasting cultivars, such as little branched and highly branched cultivars, were determined in axillary and apical buds using real-time PCR assays. These collective data describe the sequence features and expression profile of each *SMAX/SMXL* gene and reveal the key players involved in the branching of chickpea and lentil. Therefore, our results provide a solid basis for further functional studies in the chickpea and lentil focused on each *SMAX/SMXL* gene. Furthermore, these data provide powerful genes for use in both transgenesis and genome editing to improve the architecture of these leguminous crops.

## Materials and methods

### Chickpea and lentil sequences and features

Chickpea genome sequences and features were retrieved of the CGIAR v1 assembly and *Cicer arietinum* v1 annotation dataset (Varshney et al. 2013) from the Phytozome v13 database (Goodstein et al. 2012). Meanwhile, lentil genome sequences and features were retrieved of the Lcu.2RBY assembly and *Lens culinaris* CDC Redberry genome v2 annotation dataset (Ramsay et al. 2023) from the Pulse Crop database (Humann et al. 2019). In addition, additional features were retrieved of the USask assembly and *Lens culinaris* v1 dataset from the Phytozome database. The *A. thaliana* genome sequences were retrieved from the TAIR10 dataset (Cheng et al. 2017).

### Sequences analysis

Protein subcellular localization was predicted using LOCALIZER software (Sperschneider et al. 2017). Conserved domains in gene and protein sequences were identified using the PFAM database (El-Gebali et al. 2019), CD database (Marchler-Bauer et al. 2015), and InterPro Scan (Blum et al. 2021). Sequences were aligned using MUSCLE software (Edgar 2004) and curated by the Gblocks model, while evolutionary analyses were performed with Phylogeny.fr web service using maximum likelihood estimation (MLE) method with aLRT SH-like branch support and GTR (for nucleotide sequences) and WAG (for amino acid sequences) substitution models (Dereeper et al. 2008). *SMAX/SMXL* gene structures were displayed by the Gene Structure Display web server (Hu et al. 2014), while an unrooted evolutionary tree was inferred by the Neighbor-Joining (NJ) method (Saitou and Nei 1987) with 5000 bootstrap replicates using MEGA11 software (Tamura et al. 2021). The chromosomal localization of the *SMAX*/*SMXL* genes on chickpea and lentil genomes was generated using the MapGene2Chrom program (Jiangtao et al. 2015). Sequences of 2000 nucleotides upstream of the start codon were retrieved from the Phytozome database and submitted in the PlantCARE program to predict the *cis*-regulatory elements (Lescot et al. 2002). The conserved motifs in protein sequences were identified with the MEME Suite web server (Bailey et al. 2015). Protein–protein interaction network among SMAX/SMXL with partner proteins was predicted by the STRING database using the *Cicer arietinum* NCBI:txid3827 dataset as a reference (Szklarczyk et al. 2020).

### Protein 3D structures

SMAX/SMXL protein sequences were processed by the FASTA program from the EMBL-EBI webpage (https://www.ebi.ac.uk/Tools/sss/fasta/) for search proteins that most resemble (best score) with our query sequences. This tool performed a local heuristic search by sequence similarity from a protein or nucleotide databases for a query protein of the same type (Madeira et al. 2019, 2022). EMBL-EBI webpage produced the folded protein structure in HTML color-coded by the predicted local distance difference test (plDDT) and generated a PAE plot. The results refer to the “best model fit”, which represents 3D structures according to the plDDT, using a scale that goes from 0 to 100%. This parameter indicates an estimate of how the predicted structure agrees with an experimentally determined structure (Tunyasuvunakool et al. 2021). For pair-to-pair comparison, 3D structures were processed by the MODELLER v10.4 program (Webb and Sali 2016). The previous selection was based on root mean square deviation (RMSD) set to a maximum limit of 10 Angstroms (Å).

### Meta-analysis from RNAseq datasets

For tissue-specific expression in the chickpea, RNAseq datasets used in the meta-analysis were generated as reported by Jain et al. (2022) from *Cicer arietinum* cultivar ICC 4958 growth under room and field conditions. Thirty-two tissue samples representing different organs and developmental stages in at least three independent biological replicates were collected and analyzed. The expression level of each gene was FPKM normalized. In addition, for lentil tissue-specific expression, RNAseq datasets used in the meta-analysis were generated from *Lens culinaris* cultivar Cassab grown in a glasshouse at 22 °C with a 16 h photoperiod as described by Sudheesh et al. (2016). Different tissue samples were harvested from four-week-old plants using three biological replicates. Tissue-specific RNAseq datasets were normalized by log-transformed counts using the 75th percentile method. On the other hand, the RNAseq datasets used for meta-analysis from chickpea plants under abiotic and biotic stress were: (i) root tissue under drought stress experiment I and shoot tissue under drought stress (Mashaki et al. 2018); (ii) root tissue under salinity stress experiment I and root tissue under drought stress experiment II (Garg et al. 2016); (iii) before-flowering root tissue under heat stress, before-flowering leaf tissue under heat stress, after-flowering root tissue under heat stress, and after-flowering leaf tissue under heat stress (Kudapa et al. 2023); (iv) flower tissue under salinity stress (Kaashyap et al. 2022); (v) root tissue under salinity stress experiment II and shoot tissue under salinity stress (Kumar et al. 2021b); and (vi) root tissue under drought stress experiment III (Kumar et al. 2019). In contrast, RNAseq datasets used from lentil were: (i) seedling tissue infected by ascochyta blight from resistant cultivar and seedling tissue infected by ascochyta blight disease from susceptible cultivar (Khorramdelazad et al. 2018); (ii) seedling tissue under heat stress from tolerant cultivar and seedling tissue under heat stress from susceptible cultivar (Singh et al. 2019a); (iii) seedling tissue under heat stress (Sohrabi et al. 2022); (iv) seedling tissue infected by dry root rot disease (Mishra et al. 2021); (v) root tissue under alkalinity stress from tolerant cultivar and root tissue under alkalinity stress from susceptible cultivar (Singh et al. 2022a); (vi) root and leaf tissues under drought stress (Morgil et al. 2019); (vii) leaf tissue under drought stress from tolerant cultivar, leaf tissue under drought stress from susceptible cultivar, leaf tissue under heat stress experiment I from tolerant cultivar, leaf tissue under heat stress experiment I from susceptible cultivar, leaf tissue under salinity stress from tolerant cultivar, leaf tissue under salinity stress from susceptible cultivar, leaf tissue under alkalinity stress from tolerant cultivar, and leaf tissue under alkalinity stress from susceptible cultivar (Singh et al. 2022b); (viii) root tissue under salinity stress from tolerant cultivar, shoot tissue under salinity stress from tolerant cultivar, root tissue under salinity stress from susceptible cultivar, and shoot tissue under salinity stress from susceptible cultivar (Singh et al. 2021); and (ix) leaf tissue under heat stress experiment II from tolerant cultivar and leaf tissue under heat stress experiment II from susceptible cultivar (Kumar et al. 2021a). Heatmaps were generated by the SRplot web server (https://www.bioinformatics.com.cn/srplot) using log2 fold change values (stress treatment/control treatment).

### Contrasting cultivars and plant materials

The chickpea cultivars Blanco lechoso and FLIP07-318C, and lentil cultivars Castellana and Campisi were previously selected among several other cultivars under greenhouse conditions as being the most contrasting in terms of plant branching (data not shown). The phenotypic analysis to define these contrasting cultivars was carried out determining the number of branches per plant at certain times after seed germination. In order to characterize the branching of these four selected cultivars, at least 15 plants (three replicates with five plants each) of each cultivar were evaluated, and branches were counted at 25- (stage I) and 40-day-old (stage I) plants. For this, seeds were superficially sterilized with 1.5% sodium hypochlorite solution, washed abundantly with distilled water, soaked for 5 min in distilled water, and germinated in Petri dishes containing humid filter paper for three days at room temperature. Germinated seeds with a 1–2 cm radicle were transferred to pots containing commercial substrate and kept in a greenhouse at room temperature.

### RNA and gene expression

Axillary and apical buds were collected from 20-day-old plants. Total RNA was isolated with GenUP™ Total RNA kit (Biotechrabbit, Volmerstraße, Berlin, Germany) and RNA integrity was checked in agarose electrophoresis. RNA samples were treated with RNase-free RQ1 DNase I (Promega) and used for cDNA synthesis using oligo-(dT)_20_ primer and SuperScript III RT mix (Life Technologies, Carlsbad, CA, USA). The cDNA samples were diluted 1:10 (v:v) and real-time PCR assays were performed in QuantStudio 7 Flex Real-Time PCR system (Applied Biosystems, Waltham, MA, USA). The PCR mix consisted of 3 µL cDNA, 0.1 µM gene-specific primers (Suppl. Table S1), and SYBR Green PCR Master mix (Applied Biosystems). Relative expression was calculated with the 2^-∆Ct formula using *CaCAC* (Reddy et al. 2016) and *LcTUB* (Sinha et al. 2019) as reference genes for normalization (Suppl. Table S1). The *CaG6PD*, *CaTIP41*, *LcRPL2*, and *LcRBC1* reference genes were also tested with a reduced number of samples, but *CaCAC* and *LcTUB* were considered more stable in our samples. Three biological replicates for each treatment and ten plants for each biological replicate were used. All cDNA samples were carried out in technical triplicate reactions. Target-specific amplification was confirmed by the occurrence of a single peak in the melting curve. Expression data were statistically evaluated using the SASM-Agri software (Canteri et al. 2001) while heatmaps were generated by the SRplot web server.

## Results

### Basic features of the *SMAX/SMXL* family members identified in the chickpea and lentil

**Fig. 1:**
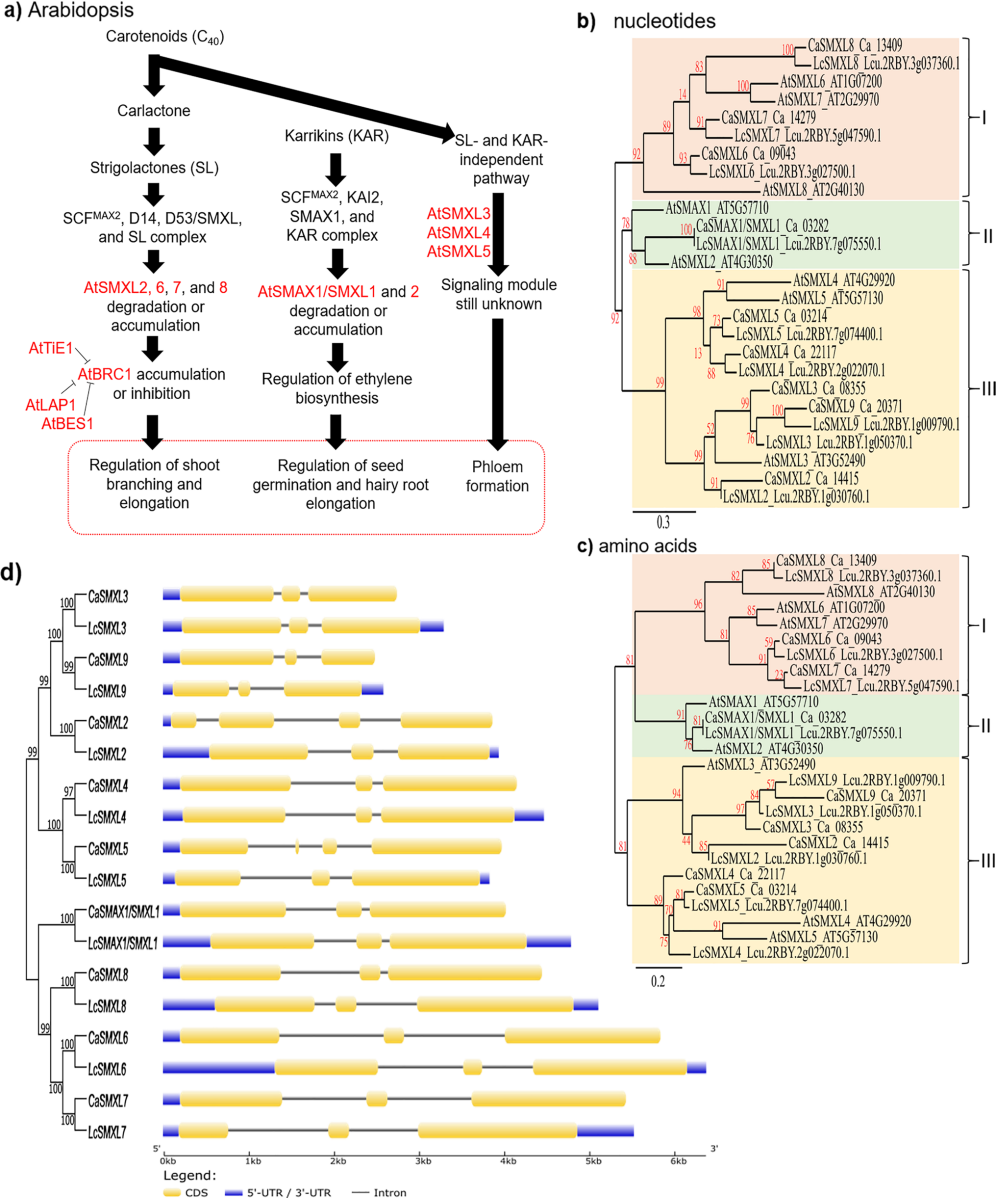
Identification and characterization of the *SMAX*/*SMXL* family genes in the chickpea and lentil using previously characterized *A. thaliana* genes as reference. **a** Overview of involvement of the AtSMXL2, AtSMXL6, AtSMXL7, and AtSMXL8 proteins in the strigolactones (SL) signaling pathway of Arabidopsis. Role of the AtSMAX1/SMXL1 and AtSMXL2 proteins in the karrikin (KAR) signaling pathway of Arabidopsis. Role of the AtSMXL3, AtSMXL4*,* and AtSMXL5 proteins in the SL- and KAR-independent signaling pathway of Arabidopsis, a module not yet well characterized. AtTiE1, AtLAP1, AtBES1, and AtBRC1 proteins are also involved in the regulation of Arabidopsis branching. These three signaling pathways mediated by SMAX/SMXL proteins were previously proposed by Soundappan et al. (2015), Carbonnel et al. (2020), Villaécija-Aguilar et al. (2019), and Wallner et al. (2017). **b** and **c** Identification of the *SMAX*/*SMXL* genes in chickpea and lentil genomes using as reference *SMAX*/*SMXL* orthologous genes of Arabidopsis. Unrooted evolutionary trees were generated from nucleotide and amino acid sequences using the MLE method. **d** Basic structure of the *SMAX*/*SMXL* genes. The unrooted evolutionary tree was inferred using the NJ method

To identify the candidates *SMAX/SMXL* family genes in the chickpea and lentil genomes were used as reference the coding (CDS) and amino acid sequences of the orthologous *AtSMAX/SMXL* genes previously identified in *A. thaliana*. The biological importance of the AtSMAX/SMXL proteins in the SL- and KAR-dependent or SL- and KAR-independent signaling pathways for *A. thaliana* was previously proposed by Soundappan et al. (2015), Carbonnel et al. (2020), Villaécija-Aguilar et al. (2019), and Wallner et al. (2017), and used in this study as information support for chickpea and lentil (Fig. 1a). The in silico analyses allowed identify nine genes in the chickpea (*CaSMAX1/SMXL1* to *CaSMXL9*) and other nine genes in the lentil (*LcSMAX1/SMXL1* to *LcSMXL9*) genomes (Tables 1 and 2). Phylogenetic relationships from nucleotide and amino acid sequences were used to define the orthologous and their corresponding genes in the chickpea and lentil (Fig. 1b and c). In particular, CaSMAX/SMXL proteins showed predicted subcellular localization at chloroplast or nucleus, predominantly with PF02861, PF07724, IPR023150, IPR027417, IPR004176, IPR003959, and KOG1051 as main domains of the PFAM, InterPro, and KOG, respectively. The *CaSMAX/SMXL* coding sequences ranged between 1866 to 3252 nucleotides in length, and deduced protein sequences ranged from 622 to 1084 amino acids in length. In addition, molecular weight (Mw) ranged between 70.4 to 120.4 kDa, and isoelectric point (pI) ranged from 5.8 to 8.2 (Table 1). In contrast, LcSMAX/SMXL proteins showed predicted subcellular localization in the chloroplast, mitochondria, and nucleus, predominantly with PF02861, PF07724, IPR023150, IPR027417, IPR004176, IPR003959, and KOG1051 as main domains of the PFAM, InterPro, and KOG, respectively. The *LcSMAX/SMXL* coding sequences ranged from 1716 to 3246 nucleotides in length, and deduced protein sequences ranged from 572 to 1082 amino acids in length. In addition, molecular weight ranged from 64 to 120.5 kDa, while isoelectric point ranged from 5.7 to 7.5 (Table 2). The detailed features concerning the *SMAX/SMXL* chickpea and lentil genes, such as gene identifiers, orthologous gene in Arabidopsis, subcellular localization, conserved domains in protein sequences, chromosomal localizations of these genes, coding and amino acid sequences length, protein molecular weight, and isoelectric point are systematically listed in Tables 1 and 2. Therefore, these data showed that the *SMAX/SMXL* family genes of chickpea and lentil have several conserved features among them, indicating that they can act redundantly in some functions, while other features are specific to some members, also suggesting role specificity for these members.

**Table 1:** Sequence features of the *SMAX*/*SMXL* genes identified in the chickpea genome

Gene name	Gene ID	Orthologous	Subcellular localization	PFAM domain	CD domain	InterPro	KOG	PANTHER	Chr	CDS (nt)	Amino acid	Mw kDa	pI
*CaSMAX1/SMXL1*	Ca_03282	*AtSMAX1*	Chloroplast	PF02861	cd19499	IPR023150	KOG1051	PTHR11638	7	3135	1045	114.5	6.6
		*AtSMXL2*	Nucleus		cl33938	IPR027417		PTHR11638:SF69					
		At5g57710			cl33329	IPR004176							
		At4g30350											
*CaSMXL2*	Ca_14415	*AtSMXL3*	Nucleus	PF02861	cl38936	IPR023150	KOG1051	PTHR11638	4	2271	757	84.7	5.8
		At3g52490				IPR027417		PTHR11638:SF69					
*CaSMXL3*	Ca_08355	*AtSMXL3*	Nucleus	PF02861	cl38936	IPR023150	KOG1051	PTHR11638	4	2484	828	88.5	7.7
		At3g52490				IPR027417		PTHR11638:SF106					
*CaSMXL4*	Ca_22117	*AtSMXL4*	Chloroplast	PF02861	cl38936	IPR023150	KOG1051	PTHR11638	1	3066	1022	114.7	6.3
		At4g29920	Nucleus			IPR027417		PTHR11638:SF124					
*CaSMXL5*	Ca_03214	*AtSMXL5*	Chloroplast	PF02861	cl38936	IPR023150	KOG1051	PTHR11638	7	2532	844	94.9	8.0
		At5g57130	Nucleus					PTHR11638:SF123					
*CaSMXL6*	Ca_09043	*AtSMXL6*	Nucleus	PF07724	cd19499	IPR003959	KOG1051	PTHR11638	5	3225	1075	120.4	6.1
		At1g07200				IPR023150 IPR027417		PTHR11638:SF68					
*CaSMXL7*	Ca_14279	*AtSMXL7*	Nucleus	PF07724	cd19499	IPR003959	KOG1051	PTHR11638	2	3252	1084	120.0	6.2
		At2g29970				IPR023150		PTHR11638:SF68					
						IPR027417							
						IPR001270							
*CaSMXL8*	Ca_13409	*AtSMXL8*	Nucleus	PF02861	cl38936	IPR023150	KOG1051	PTHR11638	5	3228	1076	119.4	6.3
		At2g40130				IPR027417		PTHR11638:SF113					
						IPR004176							
*CaSMXL9*	Ca_20371	*AtSMXL3*	Chloroplast	PF02861	cl33938	IPR023150	KOG1051	PTHR11638					
		At3g52490	Nucleus		cl38936	IPR004176		PTHR11638:SF106	4	1866	622	70.4	8.2

*Chr* chromosome, *Mw* molecular weight, *pI* isoelectric point, *PF02861* Clp_N, *PF07724* AAA_2, *cd19499* RecA-like_ClpB_Hsp104-like, *cl33938* ClpA superfamily, *cl33329* clpC superfamily, *cl38936* P-loop_NTPase superfamily, *IPR023150* Double Clp-N motif, *IPR027417* P-loop containing nucleoside triphosphate hydrolase, *PR027417* P-loop containing nucleoside triphosphate hydrolase, *IPR004176* Clp, N-terminal, *IPR003959* ATPase, AAA-type, core, *IPR001270* ClpA/B family, *KOG1051* Chaperone HSP104 and related ATP-dependent Clp proteases, *PTHR11638* ATP-dependent clp protease, *PTHR11638*:*SF69* subfamily not named, *PTHR11638*:*SF106* subfamily not named, *PTHR11638:SF124* double CLP-N motif protein, *PTHR11638:SF123* CLP amino terminal domain-containing protein, *PTHR11638:SF68* double CLP-N motif-containing P-loop nucleoside triphosphate hydrolase domain-containing protein-related, *PTHR11638:SF113* double CLP-N motif-containing P-loop nucleoside triphosphate hydrolases superfamily protein

**Table 2:** Sequence features of the *SMAX/SMXL* genes identified in the lentil genome

Gene name	Gene ID	Orthologous	Subcellular localization	PFAM domain	CD domain	InterPro	KOG	PANTHER	Chr	CDS (nt)	Amino acid	Mw kDa	pI
*LcSMAX1/SMXL1*	Lcu.2RBY.7g075550.1	*AtSMAX1*	Chloroplast	PF02861	cd19499	IPR023150	KOG1051	PTHR11638	7	3120	1040	114.5	6.5
		*AtSMXL2*	Nucleus		cl33938	IPR027417		PTHR11638:SF94					
		At5g57710			cl33329								
		At4g30050											
*LcSMXL2*	Lcu.2RBY.1g030760.1	*AtSMXL3*	Nucleus	PF02861	cl33938	IPR027417 IPR004176	KOG1051	PTHR11638	1	2493	831	93.6	5.7
		At3g52490			cl38936	IPR023150		PTHR11638:SF69					
*LcSMXL3*	Lcu.2RBY.1g050370.1	*AtSMXL3*	Nucleus	PF02861	cl33938	IPR004176 IPR023150	KOG1051	PTHR11638	1	2544	848	95.1	6.1
		At3g52490			cl38936			PTHR11638:SF106					
*LcSMXL4*	Lcu.2RBY.2g022070.1	*AtSMXL4*	Nucleus	PF02861	cl33938	IPR004176	KOG1051	PTHR11638	2	2955	985	110.4	6.3
		At4g29920			cl38936			PTHR11638:SF124					
*LcSMXL5*	Lcu.2RBY.7g074400.1	*AtSMXL5*	Chloroplast	PF02861	cl33938	IPR027417 IPR001715 IPR023150	KOG1051	PTHR11638	7	2481	827	92.8	7.5
		At5g57130			cl38936			PTHR11638:SF123					
*LcSMXL6*	Lcu.2RBY.3g027500.1	*AtSMXL6*	Nucleus	PF07724	cd19499	IPR003959 IPR027417	KOG1051	PTHR11638	3	3240	1080	120.5	6.1
		At1g07200						PTHR11638:SF68					
*LcSMXL7*	Lcu.2RBY.5g047590.1	*AtSMXL7*	Chloroplast										
		At2g29970	Nucleus	PF07724	cd19499	IPR003959 IPR001270 IPR027417	KOG1051	PTHR11638	5	2691	897	99.8	6.5
								PTHR11638:SF68					
*LcSMXL8*	Lcu.2RBY.3g037360.1	*AtSMXL8*	Chloroplast	PF07724	cl38936	IPR027417 IPR003959	KOG1051	PTHR11638	3	3246	1082	119.7	6.0
		At2g40130	Nucleus					PTHR11638:SF113					
*LcSMXL9*	Lcu.2RBY.1g009790.1	*AtSMXL3*	Mitochondria	PF02861	cl33938	IPR027417 IPR023150 IPR004176	KOG1051	PTHR11638	1	1716	572	64.0	5.7
		At3g52490	Nucleus		cl38936			PTHR11638:SF106					

*Chr* chromosome, *Mw* molecular weight, *pI* isoelectric point, *PF02861* Clp_N, *PF07724* AAA_2, *cd19499* RecA-like_ClpB_Hsp104-like, *cl33938* ClpA super family, *cl33329* clpC super family, *cl38936* P-loop_NTPase super family, *IPR023150* Double Clp-N motif, *IPR027417* P-loop containing nucleoside triphosphate hydrolase, *IPR004176: Clp* N-terminal, *IPR001715* Calponin homology domain, *IPR003959* ATPase, *AAA-type, core*, *IPR001270* ClpA/B family, *PTHR11638:SF94* heat shock protein 104, *PTHR11638* ATP-dependent clp protease, *PTHR11638:SF69* subfamily not named, *PTHR11638:SF106* subfamily not named, *PTHR11638:SF124* double CLP-N motif protein, *PTHR11638:SF123* CLP amino terminal domain-containing protein, *PTHR11638:SF68* double CLP-N motif-containing P-loop nucleoside triphosphate hydrolase domain-containing protein-related, *PTHR11638:SF113* double CLP-N motif-containing P-loop nucleoside triphosphate hydrolases superfamily protein

### Phylogenetic relationships among the *SMAX/SMXL* family genes identified in different species

The evolutionary relationship among *SMAX/SMXL* family genes of chickpea and lentil in relation to orthologous genes in *A. thaliana* and *Malus domestica* were determined from unrooted phylogenetic trees constructed using 18 candidates *SMAX/SMXL* gene sequences identified in the chickpea and lentil, eight *SMXL* gene sequences identified in Arabidopsis, and ten *SMXL* gene sequences identified in *M. domestica* (Fig. 1a and b; Suppl. Fig. S1). Evolutionary relationships allowed identify the orthologous genes between chickpea, lentil, and *A. thaliana*, as well as paralogous genes within the same legume species (Fig. 1a and b). Three major groups were identified with at least 92% bootstrap support, with *SMXL6*, *SMXL7*, and *SMXL8* genes of chickpea and lentil separately grouped in group I clustered with AtSMXL6 to AtSMXL8. Meanwhile, the *SMAX1/SMXL1* genes were grouped in group II clustered with AtSMAX1 and AtSMXL2. Finally, the *SMXL2*, *SMXL3*, *SMXL4*, *SMXL*5, and *SMXL9* genes of chickpea and lentil were separately grouped in group III clustered with AtSMXL3 to AtSMXL5 (Fig. 1a and b). This same organization in three main groups was maintained when including in the phylogenetic analysis the SMAX/SMXL sequences of *M. domestica* (Suppl. Fig. S1). Therefore, the phylogenetic relationship analysis showed that the *SMAX/SMXL* family genes of different plant species clustered forming three groups closely corresponding to their biological role in the three signaling pathways (SL-, KAR-dependent, and SL- and KAR-independent).

### Structure and chromosomal localization of the *SMAX/SMXL* genes

**Fig. 2:**
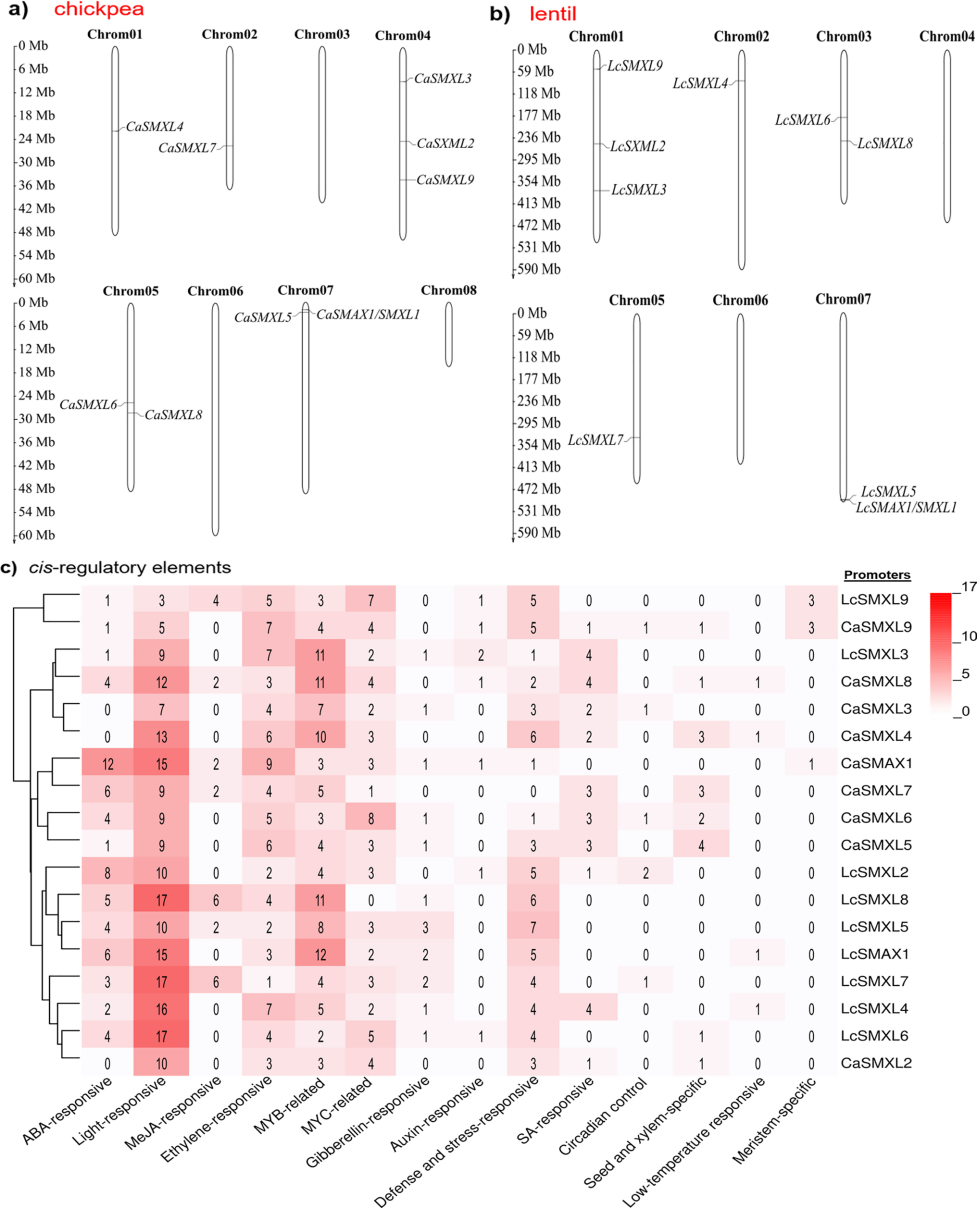
Localizations of the *SMAX*/*SMXL* genes on chromosomes of **a** chickpea and **b** lentil. Positions are based on megabases (Mb).** c** *Cis*-regulatory elements in promoter sequences (2000 nucleotides upstream of the start codon) of the *SMAX*/*SMXL* genes. Number of each *cis*-regulatory element is shown in the heatmap. *ABA* abscisic acid, *MeJA* methyl jasmonate, *MYB* MYB transcription factor binding domain, *MYC* MYC transcription factor binding domain, *SA* salicylic acid

The structural organization of the *SMAX/SMXL* family genes based on 5′-UTR, introns, exons, and 3′-UTR sequences was successfully determined (Fig. 1d). The number of introns/exons was very similar between these genes, ranging from three to four exons in each gene sequence for both chickpea and lentil. In addition, the length of the exon sequences was also similar between them, while the length of intron sequences was more variable, especially the *SMXL3* and *SMXL9* genes which included two short introns compared to *SMXL6* and *SMXL7* which showed two larger introns. The chromosomal localization of the *SMAX/SMXL* genes was also successfully determined in the chickpea (Fig. 2a) and lentil (Fig. 2b). The mapping data indicated that *CaSMAX/SMXL* genes were located on five out of eight chromosomes, one on chromosome 1 (*CaSMXL4*), one on chromosome 2 (*CaSMXL7*), three on chromosome 4 (*CaSMXL2*, *CaSMXL3*, and *CaSMXL9*), two on chromosome 5 (*CaSMXL6* and *CaSMXL8*), and two on chromosome 7 (*CaSMAX1/SMXL1* and *CaSMXL5*) (Fig. 2a). For lentil genes, the mapping data indicated that *LcSMAX/SMXL* genes were located on five out of seven chromosomes, three on chromosome 1 (*LcSMXL2*, *LcSMXL3*, and *LcSMXL9*), one on chromosome 2 (*LcSMXL4*), two on chromosome 3 (*LcSMXL6* and *LcSMXL8*), one on chromosome 5 (*LcSMXL7*), and two on chromosome 7 (*LcSMAX1/SMXL1* and *LcSMXL5*) (Fig. 2b). Based on this evidence, a similar distribution of these genes was observed between chickpea and lentil. In addition, these data may hypothesize two segmental duplication events (chromosome four and seven of chickpea and chromosome one and seven of lentil) for both plant species studied here, which eventually allowed an expansion of this *SMAX/SMXL* family.

### *Cis*-regulatory elements in SMAX/SMXL promoter sequences

Fourteen main *cis*-regulatory elements present in each promoter sequence for each of the 18 *SMAX/SMXL* genes were successfully identified (Fig. 2c). The four most prevalent *cis*-regulatory elements in these promoter sequences were light-responsive that ranged from three (*LcSMXL9*) to 17 (*LcSMXL6*, *LcSMXL7*, and *LcSMXL8*), MYB-related ranged from two (*LcSMXL6*) to 12 (*LcSMAX1/SMXL1*), ethylene-responsive ranged from one (*LcSMXL7*) to nine (*CaSMAX1/SMXL1*), and MYC-related ranged from zero (*LcSMXL8*) to eight (*CaSMXL6*). In particular, for promoter sequences of *SMXL9* genes, three *cis*-regulatory elements associated with meristem-specific expression were identified, implying that these genes may develop an important role in the early stage of plant tissue development. Similarly, promoter sequences of *CaSMAX1/SMXL1* and *LcSMXL2* genes showed twelve and eight ABA-responsive *cis*-regulatory elements, respectively. In addition, several *SMAX/SMXL* genes showed *cis*-regulatory elements responsive to gibberellin, auxin, salicylic acid, and methyl jasmonate indicating that they can be modulated by other hormones (Fig. 2c). Furthermore, although these genes showed close evolutionary conservation (Fig. 1b and c), a considerable difference in the type and number of *cis*-regulatory elements were observed (Fig. 2c), indicating a possible variable transcriptional modulation among each gene of this family and also between chickpea and lentil. Therefore, these data suggested that *SMAX/SMXL* family genes may have their expression modulated by different hormones or influenced by abiotic and biotic stress conditions, and activated primarily in a tissue or plant stage-specific manner. Thus, these data suggested that *SMAX/SMXL* family genes of chickpea and lentil can play an important role in the modulation of plant growth and development and can be modulated by different abiotic and biotic stresses.

### Conserved motifs and protein–protein interaction network of the SMAX/SMXL proteins

**Fig. 3:**
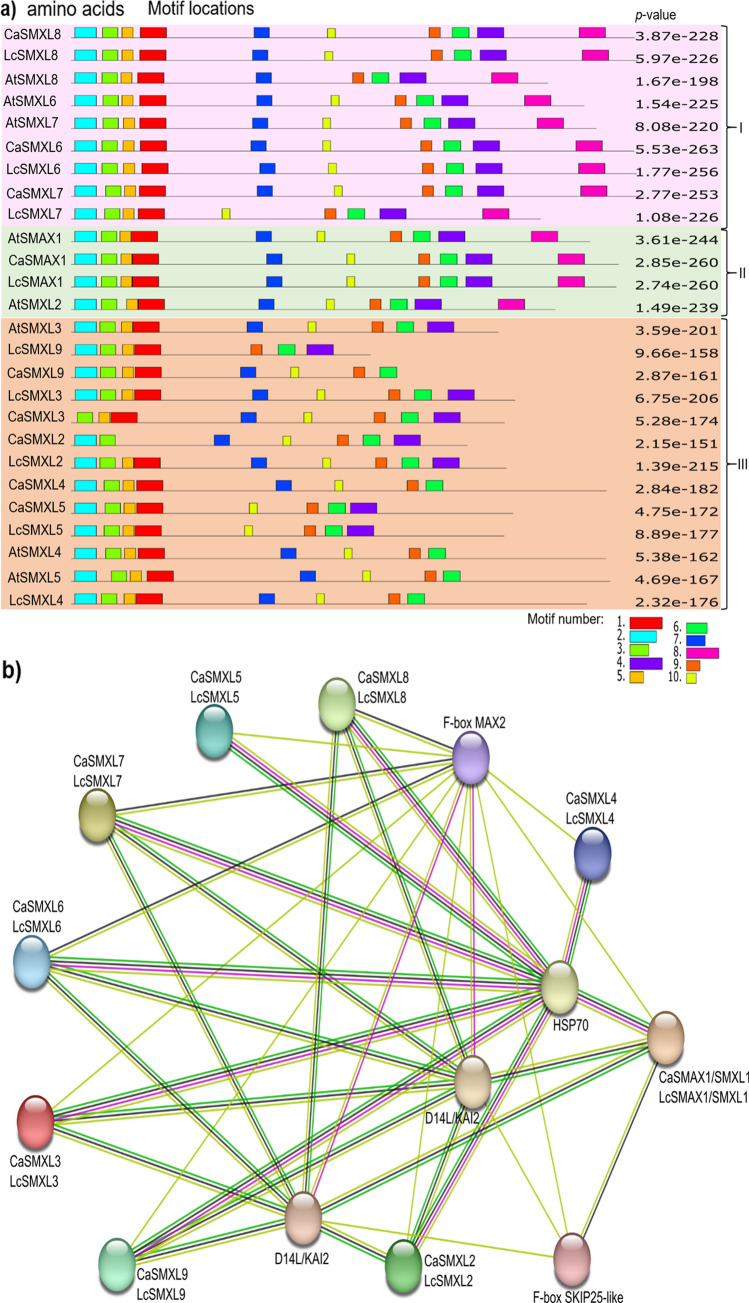
Conserved motifs and protein–protein interaction network among SMAX/SMXL with partner proteins. **a** Top 10 conserved motifs in SMAX/SMXL protein sequences of chickpea and lentil. The unrooted evolutionary tree was generated from amino acid sequences using the MLE method. **b** Protein–protein interaction network predicted by the STRING database using *Cicer arietinum* dataset as reference. HSP70: Ca_07617 and Lcu.2RBY.3g044220.1; D14L/KAI2: Ca_09326, Ca_02196, Lcu.2RBY.5g006180.1, and Lcu.2RBY.L022510.1; F-box MAX2: Ca_19880 and Lcu.2RBY.4g047720.1; F-box SKIP25-like: Ca_10634 and Lcu.2RBY.5g013390.1. Known interactions are shown in light blue line: from curated databases, and pink line: experimentally determined. Predicted interactions are shown in dark green line: gene neighborhood, red line: gene fusions, and dark yellow: gene co-occurrence. Other protein–protein associations are shown in light green line: text-mining, black line: co-expression, and light blue line: protein homology

SMAX/SMXL protein sequences of chickpea and lentil were in silico evaluated to identify the top ten conserved motifs (Fig. 3a). The number and organization of these motifs were conserved among each gene in chickpea and lentil, and with considerable similarity to the orthologous in *A. thaliana*. In particular, some motifs or their position in the protein sequence were specific for certain SMAX/SMXL proteins, indicating that some of these proteins can assume particular characteristics to perform their biological function. Subsequently, a meta-analysis of the protein–protein interaction network was conducted with the STRING database for both chickpea and lentil (Fig. 3b). These data clearly showed that all SMAX/SMXL family members are highly interconnected in a major group composed of 14 proteins. Particularly in this group, D14L/KAI2, F-box MAX2, and heat shock protein 70 (HSP70, also named hypoxia up-regulated 1 protein, HYOU1) were shown to be central hub proteins together with SMAX/SMXL proteins of both chickpea and lentil. The fourth element of this major group, F-box SKIP25-like, showed to be directly related to the SMAX1/SMXL1 proteins and interconnected with D14L/KAI2 and F-box MAX2, potentially both acting in the core of the KAR-dependent signaling pathway. Therefore, these data showed that SMAX/SMXL proteins of chickpea and lentil can play quite conserved functions and, although they act in three different signaling pathways, they form protein–protein interaction networks with the same major hub proteins. The HSP70 was shown to be the main hub protein that interconnects with all SMAX/SMXL proteins.

### 3D structure of the SMAX/SMXL proteins

**Fig. 4:**
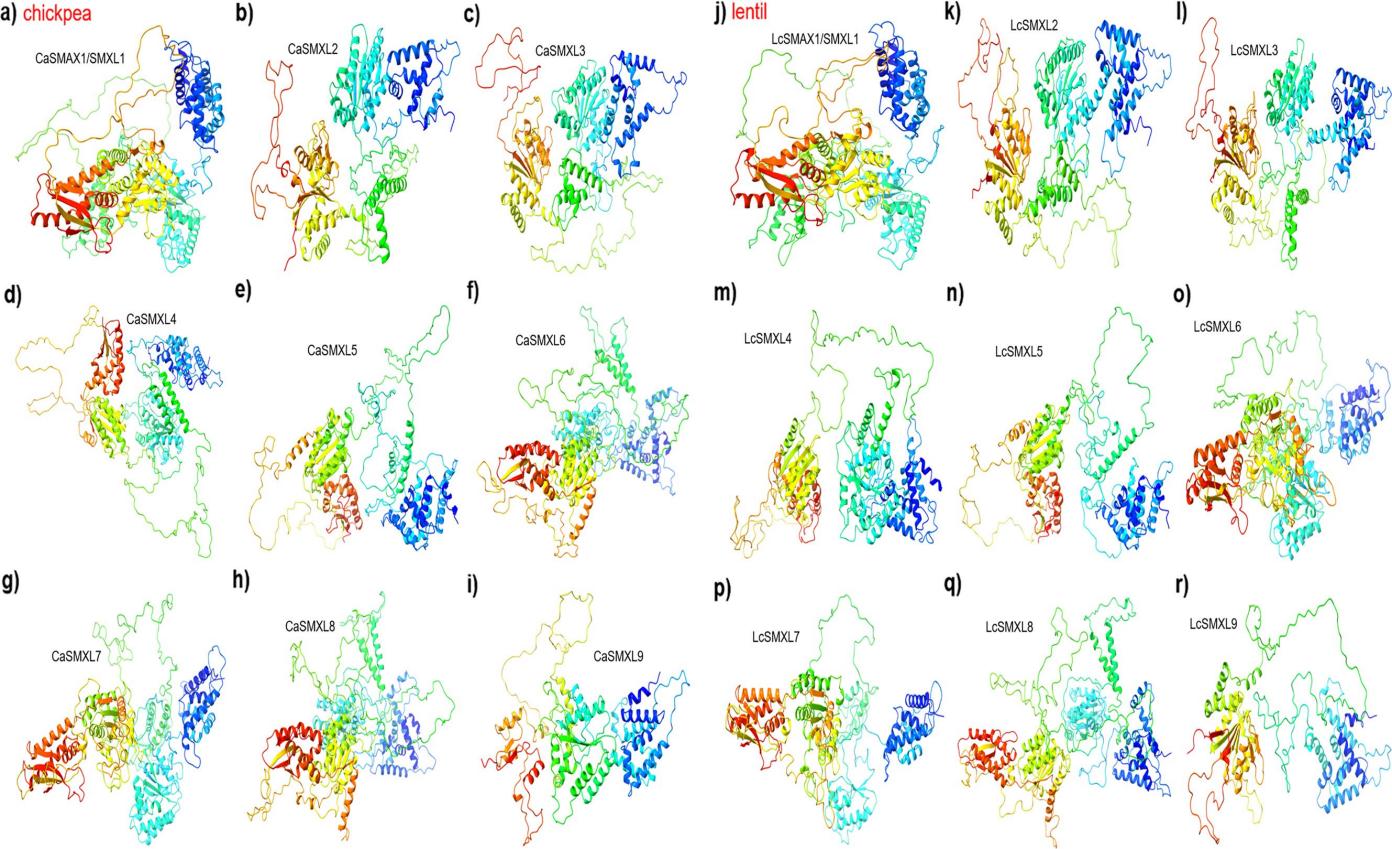
3D structure of the CaSMAX/SMXL and LcSMAX/SMXL proteins. Structure of **a** CaSMAX1/SMXL1, **b** CaSMXL2, **c** CaSMXL3, **d** CaSMXL4, **e** CaSMXL5, **f** CaSMXL6, **g** CaSMXL7, **h** CaSMXL8, **i** CaSMXL9, **j** LcSMAX/SMXL1, **k** LcSMXL2, **l** LcSMXL3, **m** LcSMXL4, **n** LcSMXL5, **o** LcSMXL6, **p** LcSMXL7, **q** LcSMXL8, and **r** LcSMXL9. Regions of different degrees of confidence are expressed with different colors according to predicted local distance difference test (plDDT) value, going from dark blue (very high confidence degree plDDT > 90%), cyan (high confident degree plDDT < 90%), yellow (relative confidence degree plDDT < 70%), and red (low confidence degree plDDT < 50%)

The 3D structures of the SMAX/SMXL proteins from chickpea and lentil were modeled and compared to each other to verify potential structural similarity. Consistently, all 18 SMAX/SMXL proteins characterized in this study had their 3D structure successfully determined (Fig. 4a–r). In addition, structural comparisons between SMAX/SMXL proteins from chickpea and lentil were performed and when similarity was greater than 66% they were considered for further analysis. The pair-to-pair structure comparison revealed 37 comparisons with a large overlapping region with > 66% homology of residues when using RMSD values maximum of 10 Å (Suppl. Table S2). These data showed that some SMAX/SMXL proteins from chickpea and lentil have considerable structural similarities and differences from each other, both between plant species or among proteins of the same plant species. Therefore, the SMAX/SMXL family has highly conserved proteins within the same species, as well as conserved proteins between different plant species, suggesting high conservation of the SL- and KAR-dependent or -independent signaling pathways in the chickpea and lentil.

### Dynamic expression of the *SMAX/SMXL* genes in different organs and under stress conditions

**Fig. 5:**
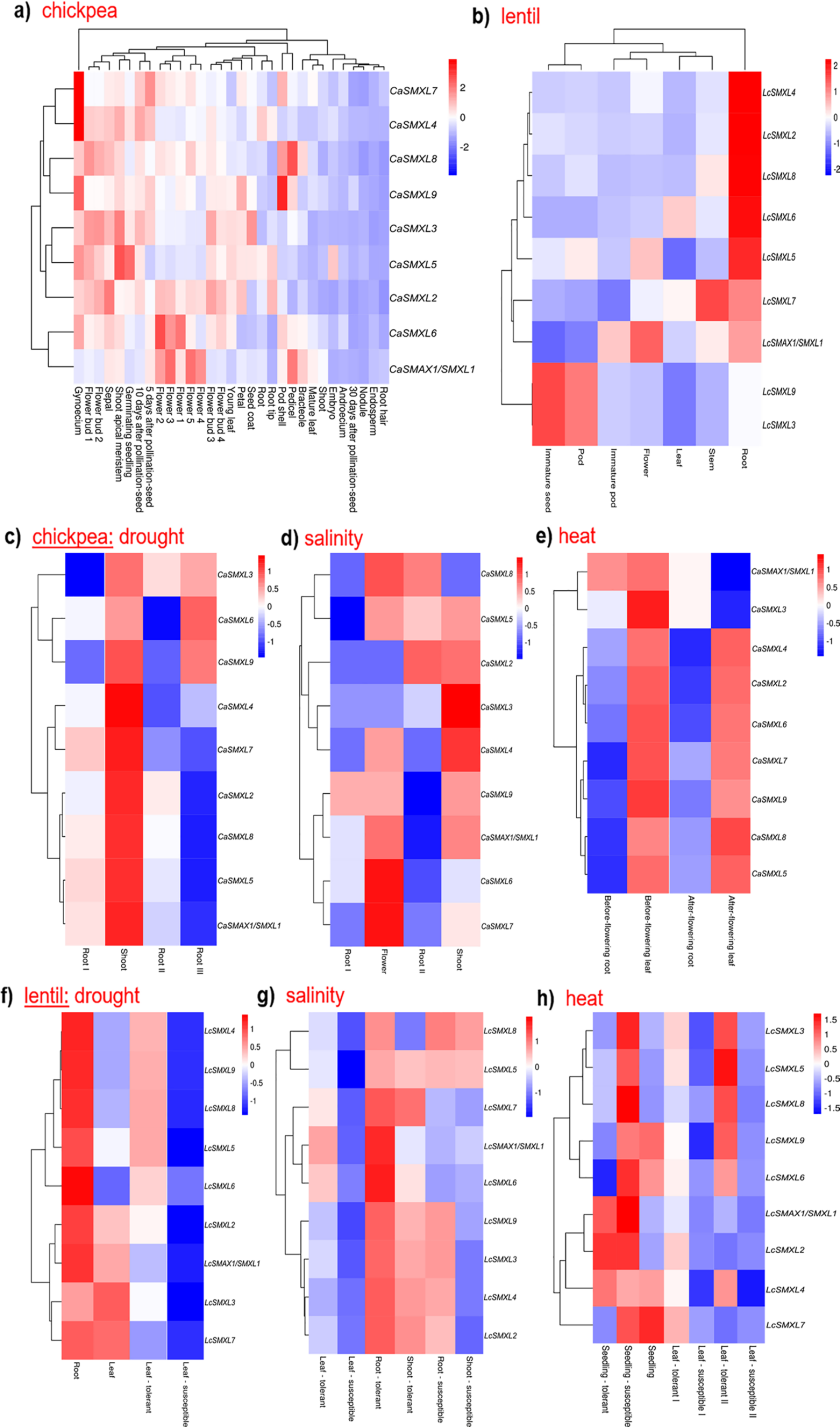
Expression profiles of the *SMAX/SMXL* genes in different organs of chickpea and lentil, and plants under different abiotic and biotic stress conditions determined from a meta-analysis using RNAseq datasets. *SMAX/SMXL* gene expression levels in different organs of **a** chickpea and **b** lentil. Gene expression in chickpea plants under **c** drought, **d** salinity, **e** heat stress conditions. Gene expression in lentil plants under **f** drought, **g** salinity, and **h** heat stress conditions. Expression values in different organs of chickpea correspond to FPKM-normalized counts for each gene, while expression values in different organs of lentil correspond to log-transformed counts using the 75th percentile method. Expression values in plants under stress conditions correspond to Log2(fold-change) contrasting “treatment *versus* control”. The scale bar indicates the expression profile

The expression profile of each of these *SMAX/SMXL* genes in different organs and in chickpea and lentil plants under different abiotic to biotic stress conditions was performed using a meta-analysis approach from RNAseq datasets. In untressed chickpea plants, it was observed that all *SMAX/SMXL* genes had their lowest expression level in the hairy root, endosperm, nodule, 30 days after pollination-seed, and androecium while the higher expression level observed was in the gynoecium (*CaSMXL7* and *CaSMXL4*), pedicel and pod shell (*CaSMXL8* and *CaSMXL9*, respectively), shoot apical meristem (*CaSMXL3* and *CaSMXL5*), and in different flowers stages (*CaSMAX1/SMXL1* and *CaSMXL6*) (Fig. 5a). In contrast, the highest expression level in lentil tissues observed was in immature seed and pod (*LcSMXL3* and *LcSMXL9*), root (*LcSMXL2*, *LcSMXL4*, *LcSMXL5*, *LcSMXL6*, and *LcSMXL8*), stem (*LcSMXL7*), and flower and immature pod (*LcSMAX1/SMXL1*) (Fig. 5b).

Meanwhile, the meta-analysis also addressed the expression profiles of the *CaSMAX/SMXL* genes in chickpea plants under drought, salinity, and heat stress conditions (Fig. 5c–e). Overall, all *CaSMAX/SMXL* genes had their expression modulated by these different stress conditions. In particular, under drought conditions, all genes had their highest expression level in shoots, while *CaSMXL6* and *CaSMXL9* genes were also significantly more up-regulated in roots (Fig. 5c). In comparison, under salinity stress conditions, the *CaSMAX/SMXL* genes were up-regulated in the flower, root, and shoot, with some genes preferentially more up-regulated in certain tissues (Fig. 5d). Meanwhile, when under heat stress conditions, all *CaSMAX/SMXL* genes had their expression up-regulated in before-flowering and after-flowering leaves, except for *CaSMAX1/SMXL1* and *CaSMXL3* genes (Fig. 5e). Likewise, the meta-analysis also addressed the *LcSMAX/SMXL* gene expression level in lentil plants under drought, salinity, heat, alkalinity, and biotic stress conditions (Fig. 5f–h; Suppl. Fig. S2a and b). In addition, this analysis also included *LcSMAX/SMXL* gene expression in contrasting lentil cultivars (tolerant, resistant, and susceptible). Similar to that observed in the chickpea, the *LcSMAX/SMXL* genes were significantly up-regulated in lentil plants under different abiotic and biotic stress conditions and showed a positive correlation with plant tolerance level to both stress conditions. In particular, under drought, salinity, and alkalinity stress conditions, the greater up-regulation of the *LcSMAX/SMXL* genes observed was in roots of tolerant cultivars compared to the leaves of these same cultivars. Interestingly, *LcSMAX/SMXL* genes are also significantly up-regulated by biotic stress and mostly correlated positively with plant resistance level (Suppl. Fig. S2b). Therefore, these collective data showed that both *SMAX/SMXL* genes of chickpea and lentil were dynamically modulated by different abiotic and biotic stress conditions, with some of these genes taking a more tissue-specific expression, and with a positive correlation with plant tolerance or resistance level.

### Expression of the *SMAX/SMXL* genes in contrasting cultivars in terms of branching

**Fig. 6:**
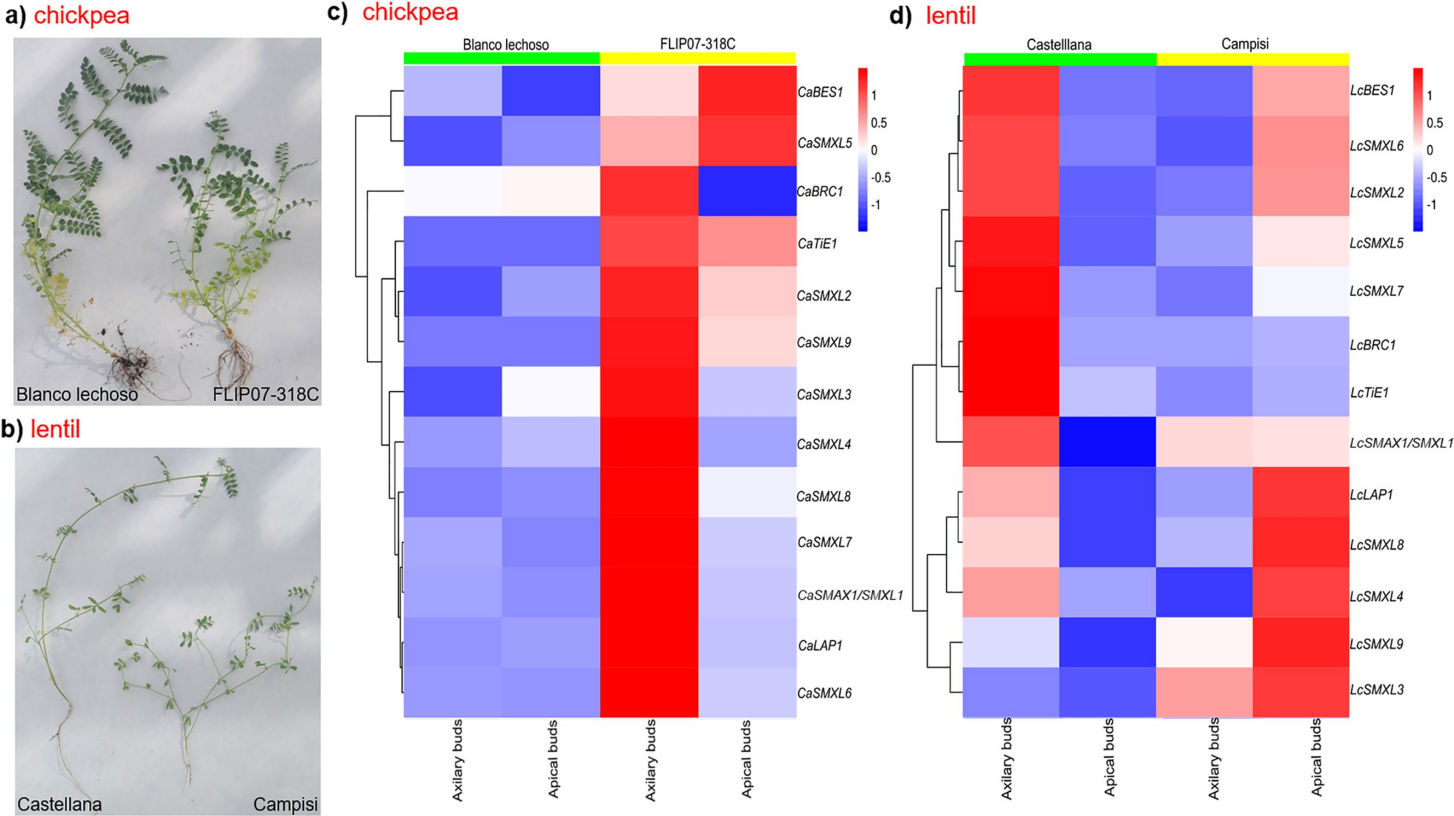
Contrasting cultivars and expression profiles of the *SMAX*/*SMXL* genes. Branching profile of the contrasting cultivars of **a** chickpea and **b** lentil. Heatmap representation displaying the expression patterns of the *SMAX*/*SMXL* genes in the **c** chickpea cultivars Blanco lechoso (little branched) and FLIP07-318C (highly branched), and **d** lentil cultivars Castellana (little branched) and Campisi (highly branched). Gene expression levels were explored by real-time PCR. The scale bar indicates the relative expression level

First, a screening was carried out with several cultivars of chickpea and lentil to identify the two most contrasting cultivars: little branched and highly branched. After phenotypic analysis based on the number of branches per plant, were selected the chickpea cultivars Blanco lechoso and FLIP07-318C, and lentil cultivars Castellana and Campisi as being little branched and highly branched, respectively (Table 3; Fig. 6a and b). During this phenotypic analysis carried out in the greenhouse, axillary and apical buds were sampled for further molecular analysis. Subsequently, the expression profiles of the *SMAX/SMXL* genes were performed by real-time PCR. In addition, *BRC1* (transcription factor involved in plant branching for acting in the SL-dependent signaling pathway), *TiE1* (TCP interactor containing EAR motif protein 1), *LAP1* (LIKE-APETALA1), and *BES1* (BRI1-EMS-SUPPRESSOR 1) genes, which encode proteins that are negative regulators of BRC1 protein, were also evaluated. For the chickpea, the highest expression level of these genes was observed in axillary buds of the highly branched cultivar (FLIP07-318C), except for *CaBES1* and *CaSMXL5* genes that showed higher expression in apical buds (Fig. 6c). These preliminary data showed that the expression profile of these genes in chickpea cultivars has a positive correlation with plant branching level. In the lentil, the expression profiles of these genes showed a lower correlation with plant branching level (Fig. 6d). In particular, the *LcSMXL3*, *LcSMXL4*, *LcSMXL8*, *LcSMXL9*, and *LcLAP1* genes showed higher expression levels in apical buds of the highly branched cultivar (Campisi), while the other genes were more expressed in axillary buds of the little branched cultivar (Castellana). These data showed that each gene or group of *SMAX/SMXL* genes, despite having several conserved features, has a slightly different expression profile between chickpea and lentil cultivars. This expression profile was also dynamic at the tissue level (axillary and apical buds), collaborating with the dynamics of the three signaling pathways in which these genes are involved.

**Table 3:** Number of branches in the chickpea and lentil cultivars kept under greenhouse conditions

Crop	Cultivar	Number of branches stage I (*n* = 3 × 5 plants)	Tukey test	Number of branches stage I (*n* = 3 × 5 plants)	Tukey test	Dominant branch	Trait
Chickpea	Blanco lechoso	2.44	a	5.65	a	Yes	Little branched
	FLIP07-318C	3.67	b	9.36	b	No	Highly branched
Lentil	Castellana	2.54	a	4.79	a	Yes	Little branched
	Campisi	5.86	c	9.86	b	No	Highly branched

Different letters indicate significant statistical differences according to Tukey’s test at a 95% significance level

**Fig. 7:**
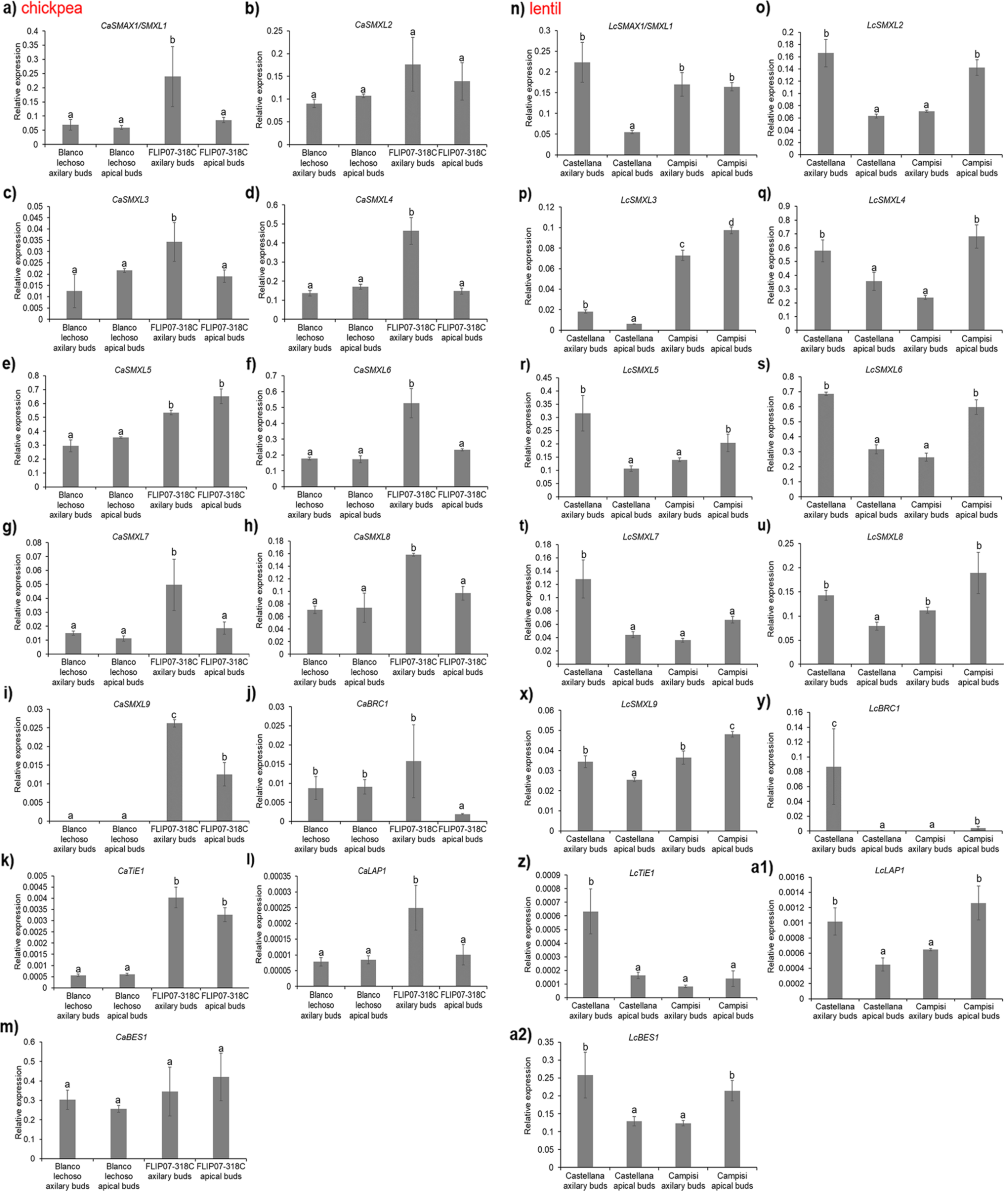
Expression profile of the *SMAX*/*SMXL* genes measured by real-time PCR in axillary and apical buds of chickpea cultivars Blanco lechoso and FLIP07-318C and lentil cultivars Castellana and Campisi. Expression profiles of chickpea **a** *CaSMAX1/SMXL1*, **b** *CaSMXL2*,** c** *CaSMXL3*, **d** *CaSMXL4*, **e** *CaSMXL5*, **f** *CaSMXL6*, **g** *CaSMXL7*, **h** *CaSMXL8*, **i** *CaSMXL9*, **j** *CaBRC1*, **k** *CaTiE1*, **l** *CaLAP1*, and **m** *CaBES1*, and lentil **n** *LcSMAX1/SMXL1*, **o** *LcSMXL1*,** p** *LcSMXL2*, **q** *LcSMXL3*, **r** *LcSMXL4*, **s** *LcSMXL5*, **t** *LcSMXL6*, **u** *LcSMXL7*, **v** *LcSMXL8*, **x** *LcSMXL9*, **y** *CaBRC1*, **z** *CaTiE1*, **a1** *CaLAP1*, and **a2** *CaBES1* genes. Gene expression values were calculated with the 2^-∆Ct formula and normalized with *CaCAC* and *LcTUB* as endogenous reference genes (Suppl. Table S1). Error bars represent confidence intervals corresponding to three biological replicates consisting of 10 plants each replicate (*n* = 10). Different letters on the bars indicate significant statistical differences according to Tukey’s test at a 95% significance level

Overall, the real-time PCR data showed that *CaSMAX1/SMXL1* (Fig. 7a), *CaSMXL3* (Fig. 7c), *CaSMXL4* (Fig. 7d), *CaSMXL5* (Fig. 7e), *CaSMXL6* (Fig. 7f), *CaSMXL7* (Fig. 7g), *CaSMXL8* (Fig. 7h), *CaSMXL9* (Fig. 7i), *CaTiE1* (Fig. 7k), and *CaLAP1* (Fig. 7l) genes were more expressed with statistical significance in the highly branched cultivar, therefore, with a positive correlation with plant branching level. Meanwhile, *CaSMXL2* and *CaBES1* genes showed no significant difference in expression level between different tissues and contrasting cultivars (Fig. 7b and m). In contrast, the *CaBRC1* gene showed lower expression with statistical significance in apical buds of the highly branched cultivar, a negative correlation with plant branching level (Fig. 7j). Meanwhile, the real-time PCR data from lentil showed that *LcSMAX1/SMXL1* (Fig. 7n), *LcSMXL2* (Fig. 7o), *LcSMXL4* (Fig. 7q), *LcSMXL5* (Fig. 7r), *LcSMXL6* (Fig. 7s), *LcSMXL8* (Fig. 7u), *LcLAP1* (Fig. 7a1), and *LcBES1* (Fig. 7a2) genes were more expressed in a tissue-specific manner, with no clear correlation with plant branching level. In contrast, the expression profiles of *LcSMXL7* (Fig. 7t), *LcBRC1* (Fig. 7y), and *LcTiE1* (Fig. 7z) genes showed a negative correlation with plant branching level, while *LcSMXL3* (Fig. 7p) and *LcSMXL9* (Fig. 7x) genes showed a positive correlation with plant branching level. Therefore, these expression data revealed that most *CaSMAX/SMXL* and partner genes are positively correlated with branching level, except the *CaBRC1* gene with a negative correlation. In comparison, the gene expression profiles in the lentil were mostly tissue-dependent, but *LcSMXL3* and *LcSMXL9* genes were positively correlated with the branching level. Furthermore, these collective data, in addition to providing new regulatory information for each gene, provide powerful targets such as *SMXL6*, *SMXL7*, *SMXL8*, *TiE1*, *LAP1*, *BES1*, and *BRC1* genes for eventual use in transgenesis and genome editing for the development of chickpea and lentil cultivars with improved architecture.

## Discussion

The increasing climate changes, eminent geopolitical conflicts, growth of the global population, and high demand for healthy food are major factors that are challenging agriculture around the world (Arif et al. 2021). Chickpea and lentil are important crops for the food security of several European and Asian countries (Landi et al. 2021; Karalija et al. 2022). The plant breeding of these legumes to improve agronomic traits associated with abiotic and biotic stress tolerance, seed yield, nutritional features, and plant architecture are important requirements to produce more at a lower cost (Basso et al. 2020, 2023; Asati et al. 2022). Particularly related to the architecture of chickpea and lentil plants, significant efforts are still needed to develop superior cultivars better adapted to mechanized planting and harvesting systems (Yang et al. 2021). Fortunately, for both chickpea and lentil there are currently available a huge amount of accessions, genotypes, and cultivars in germplasm banks worldwide that can be explored to develop these more adapted cultivars (Piergiovanni 2022; Basso et al. 2023). Although knowledge of the genetic basis associated with different agronomical traits has been explored in recent years, little is known about the molecular mechanism involved in plant branching and architecture of these two legume crops. The SL, together with other hormones, is one of the main regulators of the plant branching mechanism (Yang et al. 2020; Li et al. 2022; Zhang et al. 2022). In particular, the biological role of SL and KAR hormones in plant development and resilience to abiotic stresses has not yet been explored in detail in the chickpea and lentil. Therefore, improving knowledge about the SL and KAR signaling pathways can help to understand and develop biotechnological strategies related to plant architecture (Yang et al. 2019). In especial, *SMAX/SMXL* family genes are important players that act in the SL- and KAR-dependent and -independent signaling pathways (Wallner et al. 2017; Carbonnel et al. 2020; Wang et al. 2020; Li et al. 2022). Overall, *SMAX/SMXL* family genes are organized into three major functional groups, which correspond to the involvement of these members in the three signaling pathways described above. These three pathways act mainly on (i) shoot branching and elongation, (ii) seed germination and root elongation, and (iii) phloem formation (Soundappan et al. 2015; Wallner et al. 2017; Villaécija-Aguilar et al. 2019). These functional roles of the SMAX/SMXL proteins have been consistently studied in Arabidopsis. However, to date, *SMAX/SMXL* family genes have not yet been studied and explored in the chickpea and lentil.

In this study, were identified and characterized nine *SMAX/SMXL* family genes in the chickpea and lentil, and further analyses were performed focusing on the involvement of these genes in plant branching. The eight *SMAX/SMXL* family genes of Arabidopsis were used as a reference to successfully find the orthologous genes in chickpea and lentil genomes. In silico analyses from gene and protein sequences revealed that these members are highly conserved in the chickpea and lentil but also with some particular features. In addition, were observed the presence of the Clp-N and P-loop NTPase domains in all SMAX/SMXL proteins of chickpea and lentil, which are related to the nuclear localization and ubiquitination of these proteins, respectively (Liang et al. 2016; Khosla et al. 2020). These features indicated that the *SMAX/SMXL* genes of chickpea and lentil may range from redundant to very specific functions, similar to those observed with SMAX/SMXL family members of Arabidopsis (Carbonnel et al. 2020; Wang et al. 2020). Phylogenetic relationship data showed the organization of the *SMAX/SMXL* genes of chickpea and lentil into three major groups closely related to their putative biological function, as well as also observed with *SMAX/SMXL* genes of Arabidopsis (Soundappan et al. 2015). Similarly, other recent studies have also identified and characterized *SMAX/SMXL* family genes in different plant species, such as *M. domestica* (Li et al. 2018), soybean (Zhang et al. 2022), cotton (Jia et al. 2022), and *Populus trichocarpa* (Sun et al. 2023). In particular, in these studies were identified a variable number of *SMAX/SMXL* genes but not very different from those observed in the chickpea and lentil, except for the soybean with 31 members (Zhang et al. 2022). This higher number of *SMAX/SMXL* members in the soybean can be explained by the highly duplicated genome (Schmutz et al. 2010). Also, another important feature observed was the high conservation of these members in different plant species, thus suggesting the high importance of this protein family in green plants. Also, these data support the biological importance of SL- and KAR-dependent or -independent signaling pathways mediated by the *SMAX/SMXL* genes for seed germination, plant development, branching, and resilience. Our data also showed that segmental duplication can have occurred in the chickpea and lentil for some of these genes since some of them were highly associated with the same chromosome and relatively closely located. In the soybean, there was no tandem duplication event of the *SMAX/SMXL* genes, but 27 segmental duplication events related to 31 *SMAX/SMXL* genes were detected (Zhang et al. 2022). In *M. domestica*, duplication events of the *SMAX/SMXL* genes were also suggested as responsible for the expansion of this family (Li et al. 2018).

The promoter sequences of the *SMAX/SMXL* genes showed a considerable number of *cis*-regulatory elements associated mainly with responses to light, hormones, and defense response, while some were associated with tissue-specific expression in the chickpea and lentil. These data suggested that the expression profile of the *SMAX/SMXL* genes can be dynamically influenced in the chickpea and lentil by the plant development stage, in a tissue-specific manner, and under abiotic and biotic stress conditions. In the soybean, despite the number of *GmSMAX/SMXL* genes being approximately three times greater, the number and widespread distribution of these *cis*-regulatory elements were similar to those observed in the chickpea and lentil (Zhang et al. 2022). In the *P. trichocarpa*, light-responsive and environmental stress-related *cis*-regulatory elements were also the most abundant in their promoter sequences (Sun et al. 2023). Subsequently, the protein–protein interaction network of the SMAX/SMXL proteins of chickpea and lentil was evidenced, composed of a major group of 14 proteins, which includes five partner proteins. In particular, an HSP70/HYOU1 protein was identified as a major hub to be interconnected with all SMAX/SMXL proteins of chickpea and lentil. The HSP70/HYOU1 proved to be highly conserved in several plant species, which contain a cl17037 domain (nucleotide-binding domain of the sugar kinase/HSP70/actin superfamily). The Arabidopsis HSP70/HYOU1 protein is considered a chaperone complex protein of the endoplasmic reticulum involved in the cellular response to hypoxia and negative regulation of hypoxia-induced intrinsic apoptotic signaling pathway (Behnke et al. 2015). However, the functional relationship between HSP70/HYOU1 proteins and the SL- and KAR-dependent signaling pathway has not yet been elucidated. In addition, SMAX1/SMXL1 proteins besides showing their interaction network with D14L/KAI2, HSP70/HYOU1, and F-box MAX2, also showed an interaction network with a F-box SKIP25-like, and a KAR-related protein involved in protein ubiquitination (Nelson et al. 2010; Sepulveda et al. 2022). Therefore, these data confirmed the high relationship and conservation of functions between the SMAX/SMXL proteins of chickpea and lentil. In agreement, the 3D structure data of the SMAX/SMXL proteins revealed that these members have similar structures in the chickpea and lentil, but the small differences in the structure and composition can be important to play the different roles in SL and KAR signaling pathways. This similar pattern of high structural conservation among SMAX/SMXL proteins was also observed in *P. trichocarpa* (Sun et al. 2023).

In order to explore the expression pattern of these genes in different tissues of unstressed and stressed chickpea and lentil plants, was performed meta-analysis from RNAseq datasets. The meta-analysis data showed that *SMAX/SMXL* family genes are highly expressed at very dynamic levels in all plant tissues, indicating that they may play a remarkable role in plant growth and development. A similar tissue-specific expression pattern was observed with the *SMAX/SMXL* family genes of *M. domestica* and *P. trichocarpa* (Li et al. 2018; Sun et al. 2023). Likewise, the expression profiles of these *SMAX/SMXL* genes were also dynamic and significantly modulated in chickpea and lentil plants under different abiotic and biotic stress conditions. Similar results of *SMAX/SMXL* gene expression were observed in *M. domestica* under polyethylene glycol, abscisic acid, salinity, and cold stress treatments (Li et al. 2018). In the cotton, most *SMAX/SMXL* family genes were significantly up-regulated or down-regulated by at least one cold, heat, drought, or salinity stress condition (Jia et al. 2022). In addition, the expression profile of *SMAX/SMXL* family genes was also evaluated by real-time PCR in contrasting cultivars of chickpea and lentil in terms of branching. Especially regarding *SMAX1/SMXL1*, *SMXL6*, *SMXL7*, and *SMXL8* genes of chickpea, which are directly involved in the SL signaling pathway and act in the plant branching, showed a positive correlation with the plant branching level. Meanwhile, these same genes of lentil had an expression not clearly correlated with plant branching level. In contrast, the *BRC1* genes showed a negative correlation with plant branching levels of both chickpea and lentil cultivars. Previous studies showed that a higher expression level of *BRC1* genes is associated with reduced plant branching (Aguilar-Martínez et al. 2007). The expression profiles of the *TiE1*, *BES1*, and *LAP1* genes also revealed, at least in the chickpea, a positive correlation with plant branching level. In particular, these three proteins act as inhibitors of BRC1 protein accumulation, directly impacting plant branching (Diao et al. 2019; Hu et al. 2020; Maurya et al. 2020a). In this way, the *SMXL6*, *SMXL7*, *SMXL8*, *TiE1*, *LAP1*, and *BES1* genes are powerful targets for use in genome editing aiming a gene knockout and, consequently, developing chickpea and lentil cultivars with an improved architecture. In this context, the triple knockdown of the *AtSMXL6*, *AtSMXL7*, and *AtSMXL8* genes in Arabidopsis resulted in improved drought tolerance and reduced plant branching (Yang et al. 2020). Similarly, the knockdown of the *AtTiE1* gene in Arabidopsis also resulted in reduced plant branching (Diao et al. 2019). In the same sense, due to the fact that LAP1 and BES1 proteins inhibit BRC1, the knockout of these two genes can also result in a phenotype of reduced plant branching (Hu et al. 2020; Maurya et al. 2020a). Similarly, *BRC1* genes of chickpea and lentil are powerful targets for use in transgenesis aiming a gene overexpression and, consequently, developing chickpea and lentil cultivars with an improved architecture. The knockdown of the *AtBRC1* gene in Arabidopsis resulted in plants with highly branched, suggesting that overexpression of *BRC1* genes in the chickpea and lentil can result in reduced plant branching (Aguilar-Martínez et al. 2007; Maurya et al. 2020b). Therefore, these collective data provided new evidence to be exploited for the regulation of *SMAX/SMXL* and partner genes by transgenesis or genome editing, as well as by traditional plant breeding, to achieve genetic improvements associated with plant architecture (Basso et al. 2019).

## Conclusion

In this study, a comprehensive identification and characterization of the *SMAX/SMXL* genes from chickpea and lentil at the level of gene, protein, promotor sequence, and gene expression was systematically provided. Gene expression data revealed the expression profiles of all these genes in different organs of plants unstressed and plants under different stress conditions (abiotic or biotic), as well as in contrasting cultivars in terms of branching. These results showed that *SMAX/SMXL* genes are dynamically modulated both in the chickpea and lentil, with a positive correlation with the branching level of chickpea cultivars and a tissue-specific expression manner for the lentil. These collective data also highlighted the involvement of *SMAX/SMXL* genes in SL- and KAR-dependent and -independent signaling pathways. In addition, revealed some genes which can be interesting targets for the development of biotechnological tools based on transgenesis or genome editing to reduce plant branching and improve plant architecture. Furthermore, this study will help to understand better the biological role of *SMAX/SMXL* genes in branching and resilience to stresses of chickpea and lentil.

### Supplementary Information

Below is the link to the electronic supplementary material.

Supplementary file1 (DOCX 459 KB)

## Data Availability

All data generated were available in the manuscript as figures, tables, and supplementary tables. All gene sequences and their respective ID numbers were described in the materials and methods, and in which public and open-access databases were retrieved. All datasets used in the meta-analysis were retrieved from previously published articles and were properly cited in this work. It is also worth noting that these datasets used already had the differential expression information analyzed and normalized, so we only work with the final data.

## Abbreviations

SMAX1: SUPPRESSOR OF MAX2 1
SMXL: Suppressor of MAX2 1-Like
TiE1: TCP interactor containing EAR motif protein 1
LAP1: LIKE-APETALA1
BES1: BRI1-EMS-SUPPRESSOR 1
