# Supplementary material for: Identification and expression profile of the SMAX/SMXL family genes in chickpea and lentil provide important players of biotechnological interest involved in plant branching
Source: Planta. 2023 Nov 15;259(1):1. doi: 10.1007/s00425-023-04277-y (PMC10651550; doi:10.1007/s00425-023-04277-y)
Supplement: Supplementary file 1 — Supplementary file1 (DOCX 459 KB) [file 425_2023_4277_MOESM1_ESM.docx]

**Supplemental Table S1.** Primer sequences used in the real-time PCR.

| **Gene** | **Primer name** | **Primer sequence** | ***Tm* (^o^C)** | **Amplicon (nt)** |
| --- | --- | --- | --- | --- |
| *CaSMAX1/SMXL1* | CaSMAX1q(F) | AGGCCCAGAGCAATAGCCAA | 60 | 150 |
|  | CaSMAX1q(R) | CCATTGAAAAAGGAGTGGGAGC | 60 |  |
| *CaSMXL2* | CaSMXL2q(F) | GTGGGAGAGGTTTGCAAAAAGT | 60 | 150 |
|  | CaSMXL2q (R) | TTGTGAGTGTTGTTGTTGCTCAT | 60 |  |
| *CaSMXL3* | CaSMXL3q(F) | GTCTTCAGTTTCACTCACACCC | 60 | 139 |
|  | CaSMXL3q (R) | GCATTGGAAAGTGAAGGAGTTGA | 60 |  |
| *CaSMXL4* | CaSMXL4q(F) | AACCACAAAACCATTGGAGCAA | 60 | 94 |
|  | CaSMXL4q (R) | GGCCACCAAGGGTATGATGAA | 60 |  |
| *CaSMXL5* | CaSMXL5q(F) | GTCTCCACCAAAACAAACAGCA | 60 | 134 |
|  | CaSMXL5q (R) | TGATTGTTGGCGACGGAATCG | 60 |  |
| *CaSMXL6* | CaSMXL6q(F) | TGTGAAGGTAGGGCCTTCTACT | 60 | 103 |
|  | CaSMXL6q (R) | ATTGGTCTTTGCCAAACCCATTA | 60 |  |
| *CaSMXL7* | CaSMXL7q(F) | AGCTCCATCATCCAGTGCAG | 60 | 128 |
|  | CaSMXL7q (R) | AAGCGATGGAACTTGAGACCT | 60 |  |
| *CaSMXL8* | CaSMXL8q(F) | CGGTGCATGTGCTAACGATG | 60 | 200 |
|  | CaSMXL8q (R) | CTACACCTGGTCCCACACAC | 60 |  |
| *CaSMXL9* | CaSMXL9q(F) | CATACAGTTGCAGGCACTGAC | 60 | 83 |
|  | CaSMXL9q (R) | GTGAATGACCTCTTCTTGTTGCT | 60 |  |
| *CaBRC1*  *Ca_06609* | CaBRC1q(F) | CCAGCAAGAGAGATCGACACA | 60 | 147 |
|  | CaBRC1q(R) | ACCATTCAACTGTTTTGCTTGCT | 60 |  |
| *CaTiE1*  Ca_17893 | CaTiE1q(F) | CTCAACTTCATATGGCTTCCAACC | 60 | 123 |
|  | CaTiE1q (R) | GTGGCATTTGCATGTTCCCA | 60 |  |
| *CaLAP1*  Ca_12381 | CaLAP1q(F) | GGGAAGAGGTAGGGTTCAGTTG | 60 | 84 |
|  | CaLAP1q(R) | AGAAGTCCAGCTCTCCTTTTGG | 60 |  |
| *CaBES1*  Ca_04963 | CaBES1q(F) | TGCGAAGATATACGCGGGAT | 60 | 150 |
|  | CaBES1q(R) | ATTGCCCTTGATCCCTTGCG | 60 |  |
| *CaG6PD^*^*  *Ca_14672* | CaG6PDq(F) | ACAACGATACCAGGGTGTTACC | 60 | 116 |
|  | CaG6PDq(R) | TCTCCCATGATGCCTTTAACTC | 60 |  |
| *CaTIP41^*^*  *Ca_05619* | CaTIP41q(F) | GTTGTACTTCGGGAGAGTTGCT | 60 | 115 |
|  | CaTIP41q(R) | GGAGCTTCTGGCTTATGATGCT | 60 |  |
| *CaCAC^*^*  *Ca_02271* | CaCACq(F) | CATGGACTAGACCACCAATTCA | 60 | 110 |
|  | CaCACq(R) | AACAGTGTTGTACCCGCTCTTT | 60 |  |
| *LcSMAX1/SMXL1* | LcSMAX1q(F) | ATCAAACGAGCCATGGAGCA | 60 | 129 |
|  | LcSMAX1q (R) | AGTGCCATTGGACAGGTAGC | 60 |  |
| *LcSMXL2* | LcSMXL2q(F) | TCATCAACGTCGCGGATCAA | 60 | 127 |
|  | LcSMXL2q (R) | CCCGGCTTCTCTCATAACCC | 60 |  |
| *LcSMXL3* | LcSMXL3q(F) | TCAAGCTCTTAGCCTTGGCG | 60 | 149 |
|  | LcSMXL3q (R) | CTCCCTCGGCATTCGAAACA | 60 |  |
| *LcSMXL4* | LcSMXL4q(F) | ACGTAGAAGAGGCCATGCAC | 60 | 99 |
|  | LcSMXL4q (R) | TGGGATTTGAGACATGCGGT | 60 |  |
| *LcSMXL5* | LcSMXL5q(F) | ATAGGCTCCCAACAACACCG | 60 | 127 |
|  | LcSMXL5q (R) | TGCTGCTGCTGCTGTTCTAA | 60 |  |
| *LcSMXL6* | LcSMXL6q(F) | TGGGTTTCGATTCGGTACAGG | 60 | 133 |
|  | LcSMXL6q (R) | ATCAAAAGGTTGCGAAAACGGT | 60 |  |
| *LcSMXL7* | LcSMXL7q(F) | TCCGGCTCTAGTACATACCGA | 60 | 103 |
|  | LcSMXL7q (R) | GGCCCGAGGAGTCGTTAAAT | 60 |  |
| *LcSMXL8* | LcSMXL8q(F) | GAACCTTGCACTCCGCAAAC | 60 | 93 |
|  | LcSMXL8q (R) | CCCGACAGCAATTGGACAAG | 60 |  |
| *LcSMXL9* | LcSMXL9q(F) | GTTTCCCTCAACCGTTTGCC | 60 | 137 |
|  | LcSMXL9q (R) | GAAATACTGCGGCGTTGGTG | 60 |  |
| *LcBRC1*  Lcu.2RBY.7g064070 | LcBRC1q(F) | GCAAGCAAAACTGTGGACTGG | 60 | 125 |
|  | LcBRC1q(R) | GAAACTCCTTCACATTCAGA | 60 |  |
| *LcTiE1*  *Lcu.2RBY.2g068860* | LcTiE1.2q(F) | GCAACCACAAAGAGGATTAGGTG | 60 | 84 |
|  | LcTiE1.2q(R) | AGAGGAGGGTGAAAACCATAAGC | 60 |  |
| *LcLAP1*  *Lcu.2RBY.7g030270.1* | LcLAP1q(F) | GGGAAGAGGTAGGGTTCAGTTG | 60 | 90 |
|  | LcLAP1q(R) | TTCTTGAGAAGTCCAGCTCTCC | 60 |  |
| *LcBES1*  *Lcu.2RBY.3g070670.1* | LcBES1q(F) | AGCCCTTCTCGTATGGATGC | 60 | 134 |
|  | LcBES1q(R) | GGTGATGAGATTGGTGGCGT | 60 |  |
| *LcRPL2^*^*  *Lcu.2RBY.5g066210* | LcRPL2q(F) | ATTGAGCTGAACCCAGGACAA | 60 | 80 |
|  | LcRPL2q(R) | GACGTGGGCTCCTTCAAGATC | 60 |  |
| *LcTUB^*^*  *Lcu.2RBY.6g047660* | LcTubq(F) | CACCCAGCAAATGTGGGATT | 60 | 80 |
|  | LcTubq(R) | TGGCAGATGCGGTGAGGTA | 60 |  |
| *LcRBC1^*^*  *Lcu.2RBY.L006960*  *Lcu.2RBY.7g020650*  *Lcu.2RBY.7g020530* | LcRBC1q(F) | ATGCCTGCTCTGACCGAGAT | 60 | 80 |
|  | LcRBC1q(R) | TTTCCCCAAGGGTGTCCTAAA | 60 |  |

* Reference genes for expression normalization in the chickpea (Reddy et al. 2016) and lentil (Sinha et al. 2019).

**Supplemental Table S2.** Pair-to-pair comparison of protein 3D structures processed with homology modeling by the MODELLER program v10.4. The previous selection was based on the RMSD set to a maximum limit of 10 Angstroms (Å).

| **Protein structure 1** | **Protein structure 2** | **Percentage aligned** |
| --- | --- | --- |
| CaSMAX6 | CaSMAX7 | 90.69509831 |
| CaSMAX2 | CaSMAX9 | 88.49435728 |
| CaSMAX2 | LcSMXL3 | 87.9109659 |
| CaSMAX6 | LcSMXL6 | 84.66582598 |
| CaSMAX3 | CaSMAX9 | 83.43122859 |
| LcSMXL6 | LcSMXL8 | 81.1979786 |
| CaSMAX1 | LcSMAX1 | 79.06222721 |
| CaSMAX7 | LcSMXL8 | 78.98118403 |
| CaSMAX9 | LcSMXL9 | 78.7809648 |
| CaSMAX4 | LcSMXL4 | 78.5653166 |
| LcSMXL4 | LcSMXL5 | 77.34017219 |
| CaSMAX7 | LcSMXL6 | 77.26061966 |
| CaSMAX6 | CaSMAX8 | 76.89552239 |
| CaSMAX7 | CaSMAX8 | 76.39368896 |
| CaSMAX3 | LcSMXL3 | 76.24062835 |
| CaSMAX8 | LcSMXL6 | 76.1560476 |
| CaSMAX3 | LcSMXL2 | 75.83472454 |
| CaSMAX9 | LcSMXL3 | 75.45845597 |
| CaSMAX6 | LcSMXL8 | 74.71177184 |
| CaSMAX3 | LcSMXL9 | 74.61988304 |
| CaSMAX7 | LcSMXL7 | 74.44444444 |
| CaSMAX2 | CaSMAX3 | 74.15254237 |
| CaSMAX9 | LcSMXL2 | 73.93590386 |
| LcSMXL3 | LcSMXL9 | 72.20407137 |
| CaSMAX5 | LcSMXL5 | 71.63673331 |
| CaSMAX4 | LcSMXL5 | 70.76012908 |
| CaSMAX8 | LcSMXL8 | 70.41703414 |
| LcSMXL2 | LcSMXL3 | 70.28248588 |
| CaSMAX2 | LcSMXL2 | 70.05772006 |
| CaSMAX5 | LcSMXL4 | 69.19683882 |
| LcSMXL6 | LcSMXL7 | 69.01072706 |
| CaSMAX2 | LcSMXL9 | 68.96743668 |
| LcSMXL7 | LcSMXL8 | 68.12629205 |
| CaSMAX6 | LcSMXL7 | 67.76611694 |
| LcSMXL2 | LcSMXL9 | 67.76315789 |
| CaSMAX8 | LcSMXL7 | 66.90153903 |
| CaSMAX4 | CaSMAX5 | 66.48391813 |

**
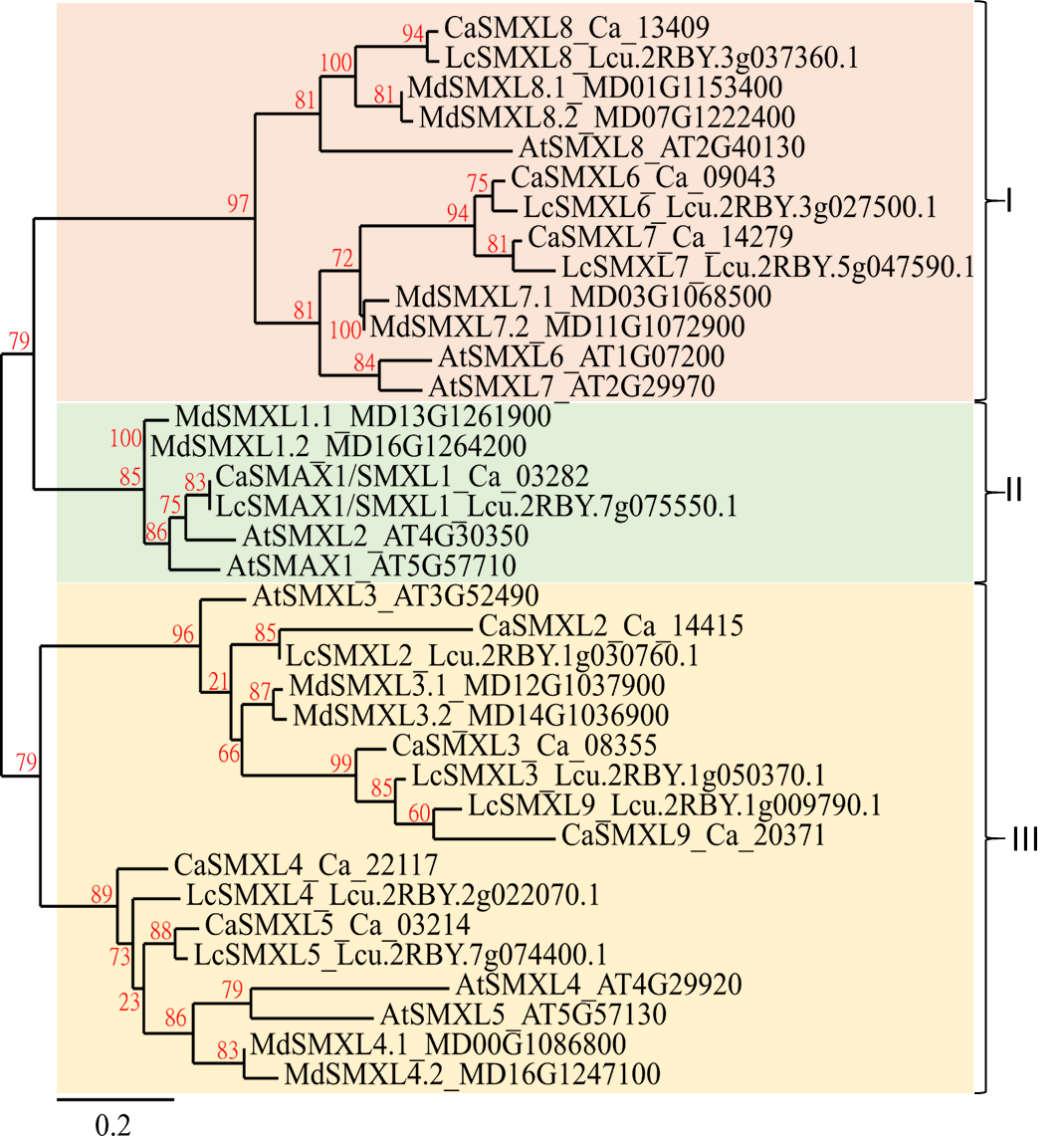
**

**Suppl. Fig. S1** Phylogenetic relationship of the *SMAX*/*SMXL* family genes identified in chickpea (Ca), lentil (Lc), *Malus domestica* (Md), and *Arabidopsis thaliana* (At) genomes. The unrooted evolutionary tree was generated from amino acid sequences using MLE method.


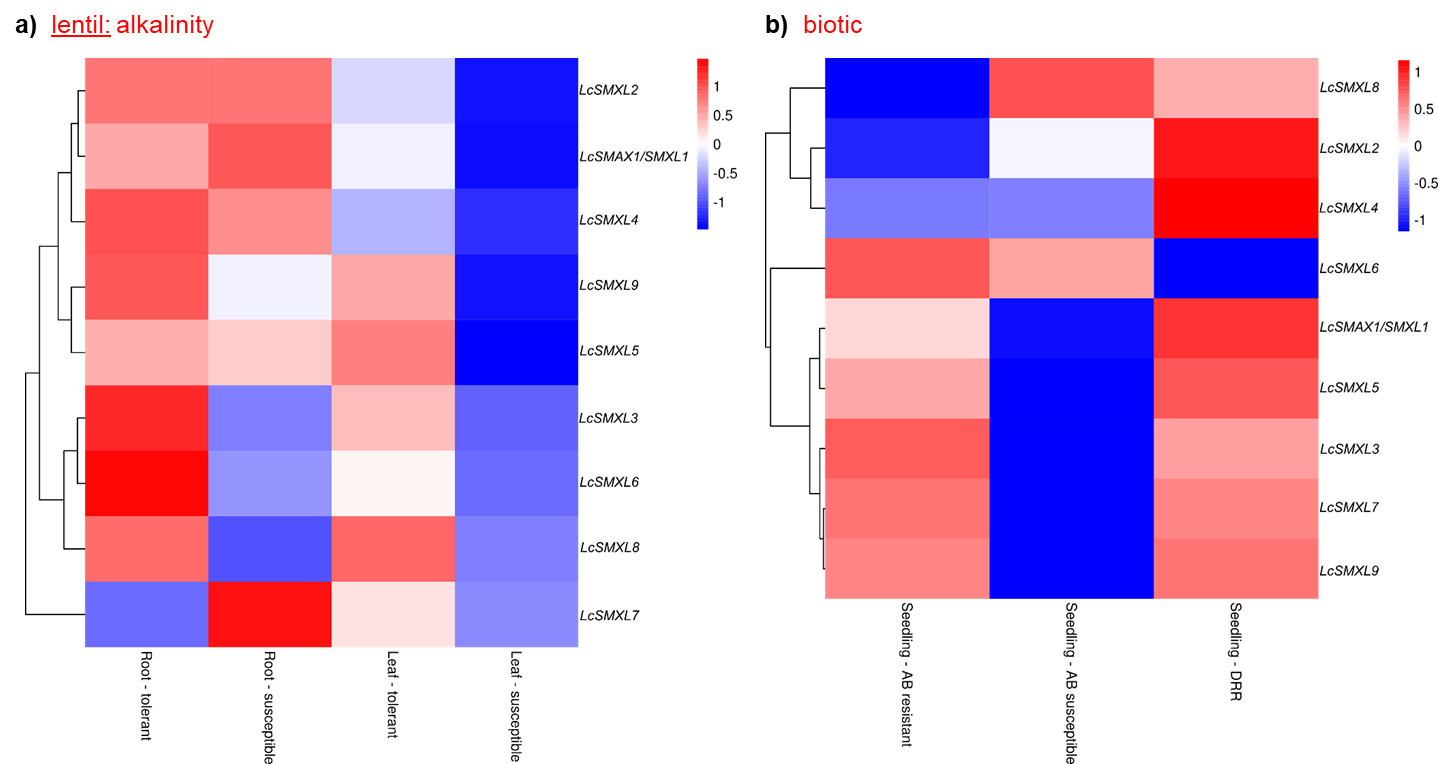


**Suppl. Fig. S2** Expression profile of the *SMAX/SMXL* genes in lentil plants under **a** alkalinity and **b** biotic stress conditions determined by meta-analysis from RNAseq datasets. AB: ascochyta blight disease, caused by *Ascochyta lentis*, and DRR: dry root rot disease, caused by *Rhizoctonia bataticola*. Expression values correspond to Log2(fold-change) contrasting treatment *versus* control. Scale bar indicates the expression profile.
